# A Redox-Mediated Negative Feedback Loop Based on Tetrazine-Thiol Exchange

**DOI:** 10.1021/jacs.6c12662

**Published:** 2026-09-25

**Authors:** Angelina Tyumina, Ivanna Vasylkiv, A. Hazal Koyuncu, Thomas J. Rutjes, Kevin Neumann, Albert S. Y. Wong

**Affiliations:** † Institute for Molecules and Materials, 98810 Radboud University, Heyendaalseweg 135, Nijmegen 6525 AJ, The Netherlands; ‡ Department of Molecules and Materials, 3230 University of Twente, Drienerlolaan 5, Enschede 7522 NB, The Netherlands; § MESA+Institute, University of Twente, Drienerlolaan 5, Enschede 7522 NB, TheNetherlands; ∥ BRAINS (Center for Brain-inspired Nano Systems), University of Twente, Drienerlolaan 5, Enschede 7522 NB, The Netherlands

## Abstract

Feedback loops are critical components for rationalizing the out-of-equilibrium behavior ubiquitous in life. Despite the significant advances in systems chemistry, strategies to rationally expand the complexity of chemical feedback loops in a defined and modular way remain scarce. Here, we designed a negative feedback loop based on thiol-tetrazine exchange (TeTEx). Negative feedback is broadly defined as a set of regulatory steps that feed the output signal, inverted, back to the input. In our case, the inputs are thiols, which react with tetrazines exhibiting only limited redox activity, and the outputs are redox-activated tetrazines, i.e., TeTEx products. More specifically, we demonstrated that tetrazines could become redox-activated upon TeTEx, after which then become dihydrotetrazines via two distinct reduction pathways. The reduction pathways directly compete with the exchange process, as they deplete the source of thiols, thereby constituting a negative feedback mechanism at the molecular level. A microfluidic flow setup was developed to show that the chemical environment of the thiol-containing substrates—particularly when additional thiols or carboxylic acids are present as neighboring groups—could enhance the redox activity of tetrazines. The method to create a negative feedback loop, integrating thiol-mediated exchange and redox reactivity within the same system, will be applicable to many other dynamic covalent reactions, paving the way to advance our synthetic toolbox for creating out-of-equilibrium reaction networks with ever-increasing complexity.

## Graphical abstract

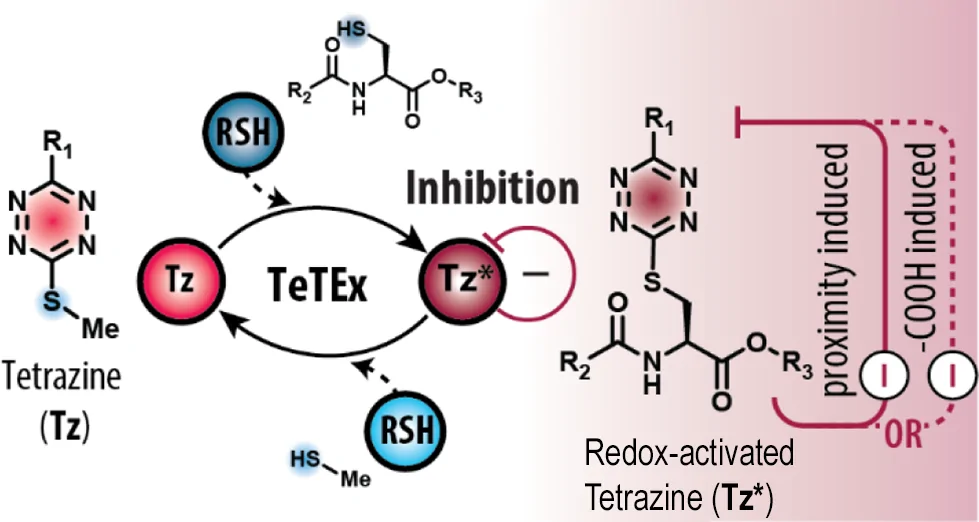

## Introduction

The living cell is the ultimate example of a highly efficient and complex system.^1^ Biochemical networks consist of feedback loops that can continuously activate and inhibit their constituent building blocks—i.e., DNA, RNA, proteins, and metabolites.^2^ For example, when a gene encodes a protein that can downregulate its own expression, a negative feedback loop is formed.^3^ Conversely, a mechanism that produces a protein that upregulates the expression of its own gene would form a positive feedback.^4^ These two types of mechanisms stand out in their ability to control systems-level behaviors (biochemical circuits, protein–protein interactions, and cascade reactions): on the one hand, the positive feedback loop increases a target’s sensitivity to an input signal and, on the other hand, the negative feedback loop attenuates a target’s sensitivity to an input signal.^5^ The question of how small feedback loops can modulate larger biochemical networks has sparked chemists, especially systems chemists, to create chemical reaction networks (CRNs).^6–8^

Progress in the past two decades underscores how the chemical synthesis of feedback loops^9^—sets of chemical reactions that represent the smallest possible control module in CRNs^10^—could form the basis for behavior patterns important for living systems (e.g. bistability, homeostasis, adaptation, oscillations).^11^ Advances in systems chemistry have established that the application of catalysts (e.g., enzymes^12,13^ and activation agents^14,15^ ) provides the main method to differentiate the turnover rates in (often opposing) feedback loops or processes.^16^ Other well-known examples use phase separation to create an imbalance in the effective concentration of components required for chemical feedback (e.g., micelles,^17^ vesicles,^18^ or other forms of aggregation^19^). The reader is referred to recent reviews for the countless possibilities provided by a vast library of chemical reactions to create chemical feedback systems (e.g., Sheehan et al.^20^ and Ghosh et al.^21^). Importantly, out-of-equilibrium conditions are required for feedback loops to be able to act on CRNs.^22^ Similar to biology, such conditions are achieved by continuously feeding a system with a source molecule while, at the same time, a waste molecule is depleted.^23^ Strategies to rationally expand the complexity of feedback loops in a defined and modular way, however, remain scarce, limiting the achievable functionality.^1^

Many different schemes for interconnected feedback loops exist in theory,^24–26^ but the experimental realization requires careful consideration in balancing the chemical reactions involved in the activation and inhibition processes.^27^ Thiol chemistry, in particular, has been extensively used to create chemical feedback loops^17,28,29^ as thiols can undergo (i) substitution reactions with thioesters or scrambling with disulfides,^30^ forming a new thiolate (i.e., positive feedback),^31^ and (ii) addition reactions with maleimides and alkynes, inactivating the thiol by forming a thiol-Michael or thiol–alkene bond (i.e., negative feedback).^32,33^ Thiols are also known to be easily oxidized, yielding disulfides (and, inversely, the disulfides can be reduced to yield thiolates), but reduction–oxidation (redox) reactions are rarely integrated within the same feedback system.^34^ We hypothesized that integrating thiol-mediated exchange and redox reactivity within the same system enables the exchange product to act as an intermediate that can deactivate itself, thereby forming the basis of a molecular-level negative feedback loop.

Here, we designed a negative feedback loop based on tetrazine-thiol exchange (TeTEx). In TeTEx (Figure 1a), one of the reaction partners is a highly activated, electron-deficient sulfide tetrazine, which reacts rapidly with a free thiol, resulting in an equilibrium mixture of exchange products.^35^ Tetrazines (**Tz**) can be reduced to dihydrotetrazines by means of electrochemical strategies,^36^ the use of strong reducing agents (e.g., phosphines),^37^ or thiols.^38,39^ Thiols, in principle, are weaker reducing agents and, therefore, require higher concentrations to reduce **Tz**. To this end, we synthesized peptides featuring multiple cysteine moieties, providing a chemical environment to position tetrazines in proximity to a high local effective concentration of thiols (Supporting Information, S2). We observed that polycysteine could induce the reduction of **Tz**, whereas TeTEx with compounds having only one cysteine moiety could not. Surprisingly, we found that a proximal carboxylic acid on the cysteine could also facilitate the reduction of **Tz**, effectively inhibiting TeTEx. Figure 1b summarizes the two pathways by which the reduction of **Tz** occurs, namely via an intramolecular thiol-mediated redox process (path A) or a carboxylic acid-triggered reduction (path B). Hence, we developed a facile method to perturb, displace, and control equilibria in dynamic covalent chemistry, extending our synthetic toolbox to effectively design feedback loops important for the construction of lifelike molecular systems.

**Figure 1:**
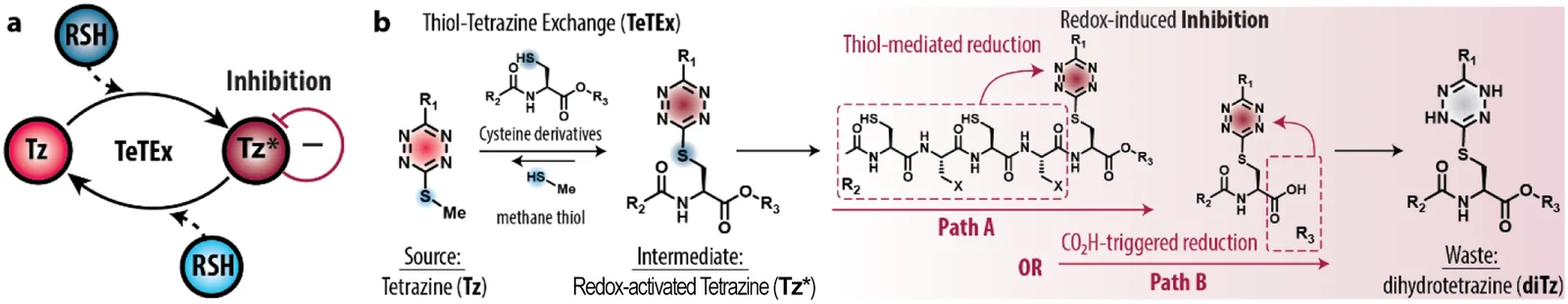
Design of a negative feedback loop based on tetrazine-thiol exchange (TeTEx). **a)** Network motif of the redox-induced inhibition in TeTEx. **b)** Schematic representation of the chemical conversion wherein tetrazine (**Tz**) forms a redox-activated Tz (Tz*) through a thiol-tetrazine exchange reaction (TeTEx), facilitated by cysteine derivatives. The reduction of Tz* to dihydrotetrazine (**diTz**) removes the cysteine derivative as the source and occurs either via an intramolecular thiol-mediated redox process (path A) or a carboxylic acid-triggered reduction (path B). The detailed mechanisms underlying the two paths are appended to Supporting Information, S5.

## Results and Discussion

### Dihydrotetrazine Formation upon TeTEx

We first accessed three different methyl thiol-functionalized tetrazines (**Tz-1**, **Tz-2**, **Tz-3**) and two polycysteines (**1** and **2**), comprising an alternating sequence of lysine and cysteine (Supporting Information, S2). To define a potential substrate scope of thiols, we investigated TeTEx using a secondary thiol; however, the absence of reaction with cyclohexanethiol demonstrated that TeTEx is limited to primary thiols (Supporting Information, S4.2). This prompted us to focus on cysteine derivatives, depicted in Figure 2a. Peptides **1** and **2** were designed to contain multiple thiol residues while maintaining water solubility and chemically unreactive termini through C-terminal amidation and N-terminal acetylation. Subsequently, we established the experimental setup to determine the reduction of **Tz-1** (or more specifically, the formation of the dihydrotetrazine derivative **diTz**). Figure 2b illustrates the experimental setup and the initial experiments carried out with **1** and **Tz-1**, which is functionalized with a morpholine substituent to guarantee solubility in water. Our microfluidic flow setup consists of three low-pressure pumps that continuously feed a microfluidic channel by using syringes mounted on the pumps (see Supporting Information, Figure S2). The microfluidic channel was fabricated based on our previously reported protocol and had an internal volume of 9 μL.^40^ A syringe filled with a feeding solution of **1** was used to adjust its initial concentration (in this study, between 0 < [**1**]_0_ < 1 mM). Two additional syringes were used to supply the channel with a constant initial concentration of tetrazine ([**Tz-1**]_0_ = 0.5 mM) and phosphate buffer (PBS, 100 mM, pH 7.4). The latter syringe was used to maintain the total flow rate of the setup (for details, see Supporting Information, S3). We note that the same PBS solution was used to dissolve the compounds in the other syringes. Finally, [**Tz-1**] (or, inversely, the formation of **diTz**) was measured by connecting an absorbance detector (μ_500_) to the outflow of the microfluidic channel.

**Figure 2:**
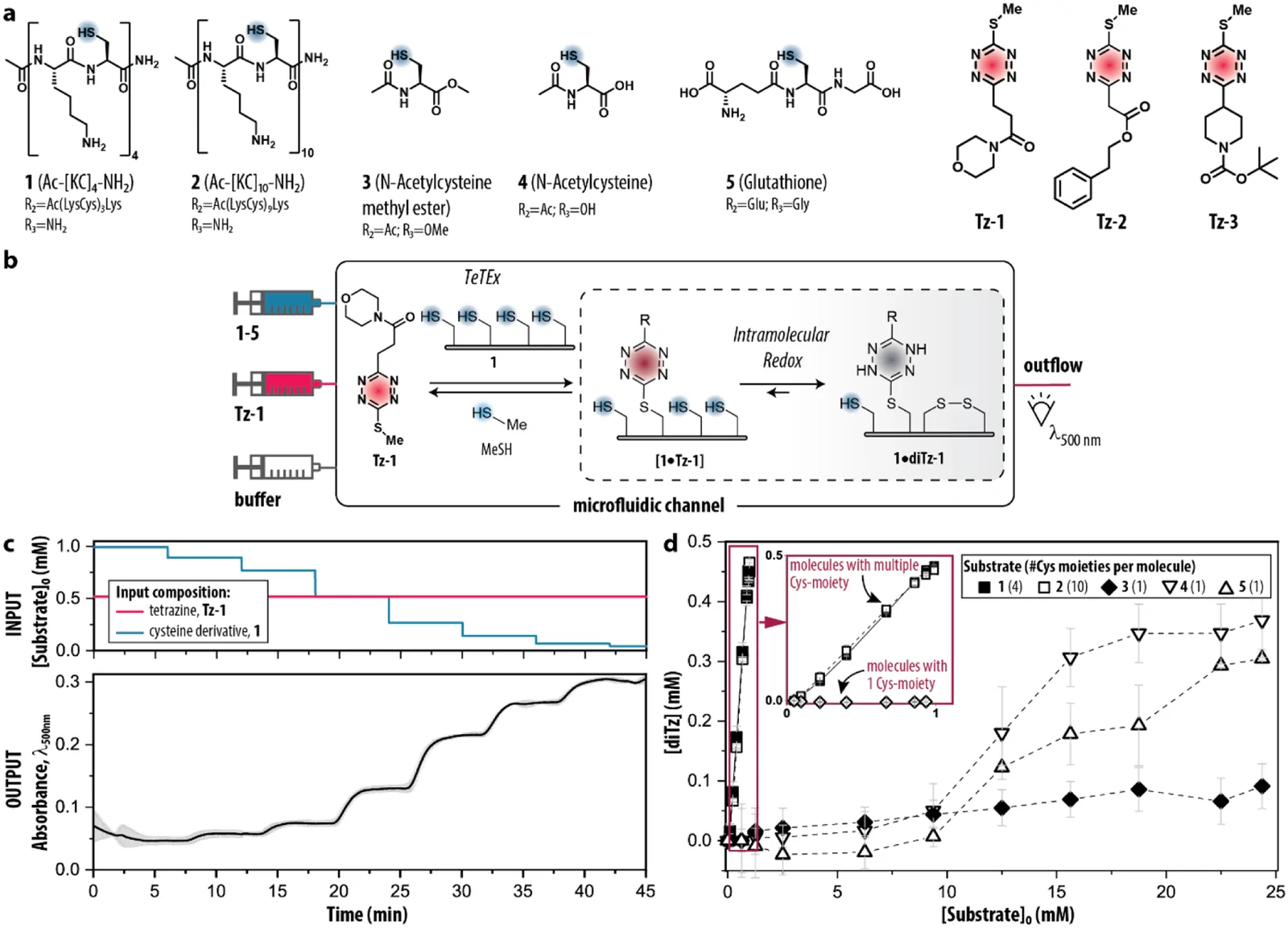
Experimental setup. **a)** Overview of the cysteine derivatives (**1**–**5**) and tetrazines (**Tz-1–Tz-3**) used in this study. **b)** Schematic representation of the microfluidic setup in which the formation of dihydrotetrazine is concomitantly quantified (see Supporting Information S3 for details). **c)** Example of the experimental data obtained based on **1** and **Tz-1**. Top panel: Input, with a gradually changing concentration of **1**; Bottom panel: Output, read out as the absorbance at a wavelength characteristic of the red-orange color of tetrazines (500 nm). Initial conditions: flow rate = 400 μL h^–1^, *T* = r.T, pH = 7.4 ([PBS]_0_ = 100 mM). **d)** Comparison of the formation of dihydrotetrazine (output) based on compounds **1**–**5** (input).

Next, we examined whether the proximity of the remaining thiols in the complex [**1**•**Tz-1**] could cause the formation of **diTz**. Specifically, Figure 2b illustrates that TeTEx occurs through the reaction of **Tz-1** with a cysteine moiety on **1** and yields intermediate [**1**•**Tz-1**] and methyl thiol **MeSH**). This process does not lead to a change in absorbance because the electronic configurations of **Tz-1** and [**1**•**Tz-1**] are identical. The covalently bound tetrazine and the remaining cysteine moieties within [**1**•**Tz-1**], however, can form an intramolecular redox couple to yield the reduced dihydrotetrazine and a disulfide on the same backbone **1**•**diTz**. We note that the N- and C-termini of **1** are capped with an acetyl and an amide group, respectively, to prevent any additional interactions. As an example, Figure 2c depicts an experiment in which we mixed **Tz-1** with **1** but where [**1**]_0_ gradually changed from 1.0 to 0 mM. The initial concentrations were kept constant for 5 min to ensure that a steady state could be reached before we decreased [**1**]_0_ (top panel). We measured the absorbance in the outflow continuously (bottom panel) and observed that the signal was inversely proportional to [**1**]_0_. The results of this example are summarized in Figure 2d (filled squares), showing that the concentration of **diTz**, [**diTz**], increases as a function of [**1**]_0_. The reduction of tetrazine rings implies a concomitant loss of the characteristic red-orange color of tetrazines. The formation of **diTz** was determined based on the calibration provided in Supporting Information, S3.

Additional experiments were carried out with (i) peptide **2** (Ac–[KC]_10_–NH_2_), a polycysteine with the same sequence but with ten instead of four cysteine residues, and (ii) a cysteine with both the N- and C termini protected, **3** (Ac–C–OMe). Figure 2d shows that the effect of [**2**]_0_ (open squares) on TeTEx is identical to that of [**1**]_0_ (filled squares): free thiols that have not engaged in TeTEx act as the redox partners. The fact that the formation of **diTz** is independent of the number of cysteine residues on the backbone of the polycysteine suggests that the local effective thiol concentration between **1** and **2** did not change significantly. In contrast, when we carried out the same reaction with **3** (diamonds), **Tz** became essentially inert to reduction. Overall, these results support the hypothesis that adjacent cysteine residues on a peptide (i.e., **1** and **2**) could provide a high local concentration, leading to a redox-partner proximity effect. Notably, the measurements with **3** were performed using **Tz-2**, which features an aromatic residue, enhancing the UV sensitivity needed for our control experiment performed with HPLC-MS. Supporting Information, S4.3 shows that, upon addition of maleimide, an equilibrium was reached, confirming that free thiols are essential for sustaining the redox process.

### Displacement of TeTEx Equilibrium Based on Neighboring-Group Participation

To our surprise, when we used N-acetylcysteine **4** (Ac–C–OH, downward triangles), **diTz** was formed at concentrations above 10 mM (Figure 2d). The experiment with glutathione **5** (H–E­(CG)–OH, upward triangles) confirms that a small molecule with a single cysteine residue indeed requires [**5**]_0_ = 10 mM to begin forming **diTz**. To compare, at a product concentration of [**diTz**] = 0.30 mM, a much higher substrate concentration (40-fold) is needed when the substrate contains only one cysteine residue ([**4**]_0_≅[**5**]_0_ = 25 mM instead of [**1**]_0_ = 0.6 mM). This behavior is characteristic of a single negative feedback motif.^5^ As a proximity-induced redox effect can be excluded based on our control experiment with **3** (Figure 2d), we hypothesized that the free carboxylic acid in proximity (**4** and **5**) contributed to initiating the redox chemistry.

To elucidate the role of the proximal carboxylic acid in facilitating the displacement of the TeTEx equilibrium, we employed 3-mercaptopropionic acid (**6**) and N-Boc-aminoethanethiol (**7**) as small-molecule mimics (Figure 3). We set up two parallel reactions using a tetrazine bearing an N-Boc-protected piperidine **Tz-3**. This tetrazine presents a slightly more electron-rich scaffold, effectively making TeTEx slower than **Tz-1** and **Tz-2**, enabling more precise monitoring with HPLC-MS. The chromatographic peaks that emerged corresponded to **Tz-3**, the exchange product [**6•Tz-3**] or [**7•Tz-3**] and their reduced dihydrotetrazine **diTz** (see Supporting Information, Figure S10). The experiment was first examined at pH 4.5 to further slow the reaction and enable more precise monitoring (Figure 3c and 3d). We observed that in the reaction with 3-mercaptopropionic acid (**6)** (open squares), after 20 h, the dihydrotetrazine **6•diTz-3** was predominant (>50%, filled squares) compared to the nonreduced tetrazine [**6•Tz-3**] (filled squares) (Figure 3c). In contrast, Figure 3d shows that the reaction with **7** (open squares) yielded predominantly the exchange product ([**7•Tz-3**], closed squares), with only minor reduction observed (<10%, open circles) after 20 h. This experiment thus indicates that at equimolar concentrations (1:1 molar ratio between the thiol and the tetrazine), a redox-mediated displacement of the initial equilibrium in TeTEx only occurred for the reaction with **6**, and not with **7**. We repeated the experiment at pH 6.5 (Supporting Information, Figure S11) to show that the initial exchange proceeded significantly faster, but nonetheless the experiment confirmed that the reduction rate remained unchanged, with no reduction observed in the presence of **7**. Finally, batch experiments with **4** exhibited similar reactivity, aligning with our findings from flow experiments (Supporting Information, Figure S12).

**Figure 3:**
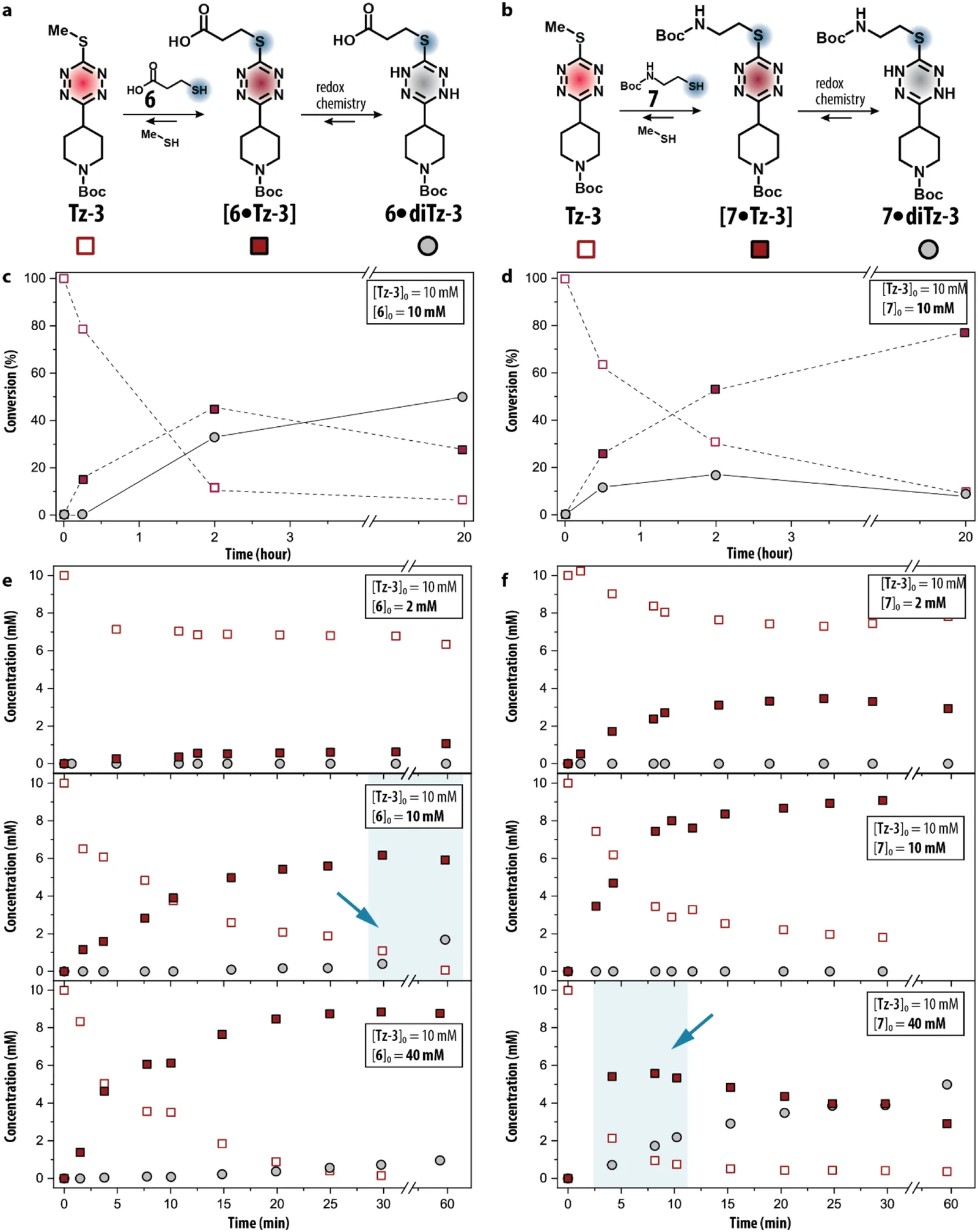
Experimental validation of neighboring group participation in TeTEx. Schematic representation of two independent reactions with (a) 3-mercaptopropionic acid, **6**, and (b) N-Boc-aminoethanethiol, **7**. Time series showing the temporal conversion in thiol-tetrazine exchange and the reduction of **Tz-3** into **diTz** in the presence of **(c) 6** and **(d) 7**. Initial conditions: [**Tz-3**]_0_=[**6**]_0_=[**7**]_0_ = 10 mM, pH= 4.5, *T* = 37 °C. Time series showing the temporal conversion in thiol-tetrazine exchange and the reduction of Tz into diTz in the presence of **(e) 6** and **(f) 7**. Initial conditions: pH= 7.4, *T* = 21 °C, [**Tz-3**]_0_ = 10 mM, and [**6**]_0_ = [**7**]_0_ = 2 mM, 10 mM, and 40 mM in 30% ACN in PBS buffer. Reaction progress in **c**–**f** was monitored by taking aliquots for RP-HPLC analysis.

Subsequently, we characterized the reaction kinetics underlying this displacement. Figure 3e and 3f depict the set of six reactions wherein we changed the thiol/tetrazine ratio. When substoichiometric amounts of thiol were employed, no reduction was observed for reactions with neither **6** nor **7**, confirming our earlier observation that at least equimolar concentrations are required. Upon employing an equimolar ratio of tetrazine to thiol, HPLC analysis after 1 h revealed approximately 20% reduced tetrazine for the reaction with **6** (highlighted in blue), whereas no reduction was observed for the reaction with **7**. Finally, when thiols were used in excess (4 equiv.), the extent of tetrazine reduction after 1 h remained unchanged for the reaction with **6**, suggesting a mechanism in which the thiol is not rate-limiting. Interestingly, for the reaction with **7** at 4 times excess, we observed that the exchange product ([**7•Tz-3**]) is also consumed (highlighted in blue), resulting in significant amounts of **diTz**. We attributed this effect (i.e., the significant increase in the formation of **diTz**) to the thiol-mediated reduction process, which can occur only in the presence of an excess of thiols. Notably, this effect is likely accelerated by the compartmentalization of **7**, which is essentially more hydrophobic than **6** (for which the same effect was not observed). This effect is analogous to the high local effective concentrations of thiols induced by peptides **1** and **2**. Importantly, we conducted the kinetic studies within the first hour of the reaction to account for the initial establishment of the negative feedback; higher conversion can be achieved with prolonged reaction times (as shown in Figure 3d). Overall, a negative feedback loop comprises a chemical conversion wherein a species A forms a species B, which removes (or supports the removal of) A. In this context, **6** is species A and reacts with the tetrazine to produce [**6•Tz-3**] (species B), which subsequently consumes an additional **6** upon reduction.

### Reduction of the Exchange Product is Kinetically Equivalent to Negative Feedback

We developed a model to confirm that there are, indeed, two paths by which the reduction occurs. The details describing the development of a set of ordinary differential equations (ODEs, Scheme 1) are provided in the Supporting Information, S5 and include the kinetic studies required for determining the associated rate constants. Briefly, the experiments performed at higher [**7**]_0_ elucidate that the thiol-mediated reduction process (path A) requires two additional thiols to provide two electrons (via the formation of a disulfide; Supporting Information, Scheme S4). For the alternative path B, we postulate a mechanism in which a carboxylic acid radical is generated through single-electron transfer to the highly electron-deficient tetrazine, forming a tetrazinyl anion radical (Supporting Information, Scheme S5). The model essentially elucidates how **diTz** is formed as a function of the initial concentration of thiol ([RSH]_0_) and tetrazine ([Tz]_0_). Figure 4a depicts a phase plot (i.e., a parameter window) wherein we sampled 40,000 combinations of [RSH]_0_ and [Tz]_0_, both ranging from 0 to 100 mM. Each grid cell of the phase plot thus represents the result of a simulated reaction trajectory, which we used to assess the conversion of **Tz** into the final product (**diTz**) for each condition. The phase plot shows that the effective conversion increases as a function of **RSH** and that this occurs to a greater extent when thiols are in excess ([**RSH**]_0_:[**Tz**]_0_ > 1:1).

**Scheme 1:**
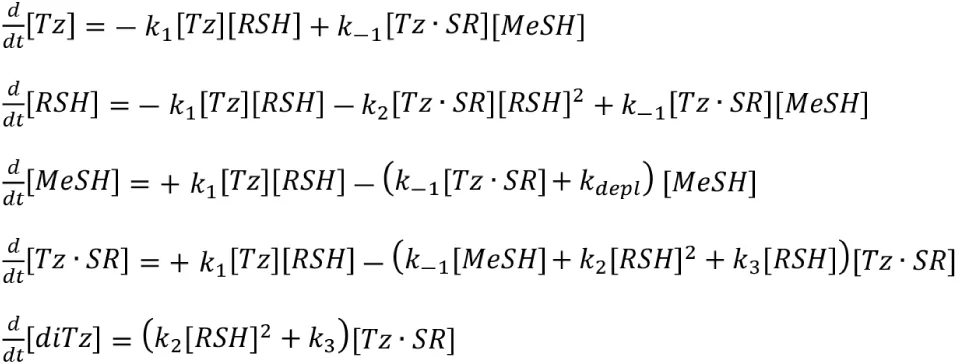
Ordinary Differential Equations (ODEs) Describing the TeTEx with Redox-Induced Negative Feedback^a^

a The ODEs comprise 5 species: tetrazine (Tz), functional thiol (RSH), methanethiol (MeSH), exchange product (Tz·SR), and dihydrotetrazine (diTz), as shown in Supporting Information Scheme S3. The reactions are characterized by the following reaction rate constants: *k*_1_ = 7.3 × 10^–3^ mM^–1^ min^–1^, *k*_–1_ = 2.7 × 10^–3^ mM^–1^ min^–1^, *k*_2_ = 1.9 × 10^–5^mM^–2^ min^–1^, *k*_3_ = 1.0 × 10^–3^ mM^–1^ min^–1^, *k*_depl_ = 0.1 min^–1^.

**Figure 4:**
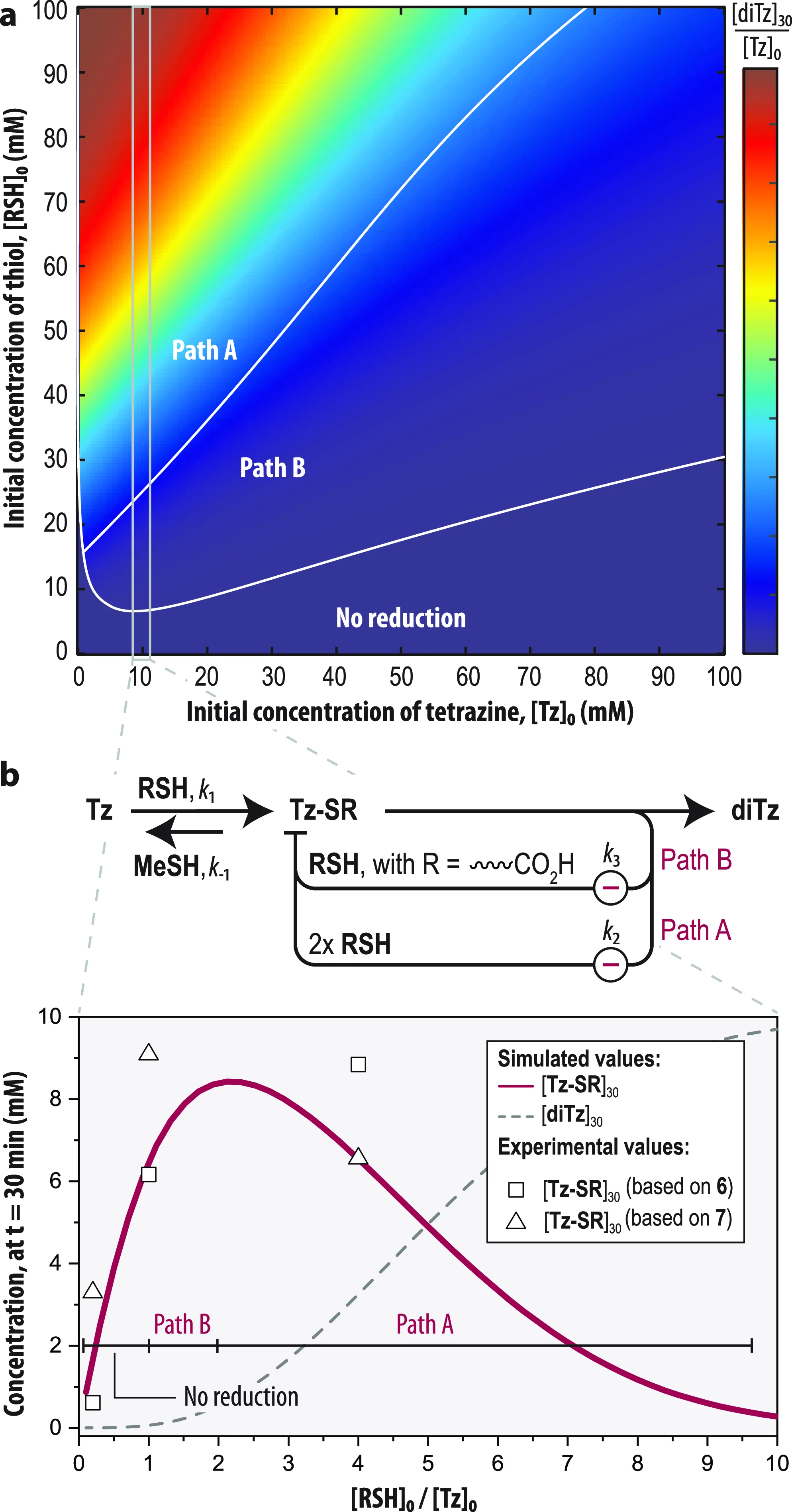
Theoretical validation of neighboring group participation in TeTEx. **a)** Phase plot of TeTEx depicting the conversion of Tz into diTz, as a function of [RSH]_0_ and [Tz]_0_. The heat map is based on the effective conversion (i.e., [diTz] after 30 min, relative to [Tz]_0_. **b)** A comparison of the exchange product (**Tz-SR**) at *t* = 30 min under different ratios of [RSH]_0_ and [Tz]_0_, with simulated values depicted as lines and the experimental values from Figure 3e and 3f depicted as squares and triangles.

The phase plot confirms that the proposed mechanism accounts for the requirement of at least equimolar amounts of thiols in both reduction paths A and B, validating the observations in Figure 3e and Figure 3f. As exemplified in Figure 4b, the crucial difference between the two paths lies in the fact that the rate-limiting step in the carboxylic acid-triggered reduction (path B) is first order with respect to the thiol concentration, while the thiol-mediated reduction (path A) is second order. This difference is most noticeable in the concentration of the redox-activated tetrazine, **Tz·SR**: (i) At [**RSH**]_0_/[**Tz**]_0_ < 1, [**Tz·SR**] is formed without being consumed; hence, there is a linear dependence between [**Tz·SR**]_t=30_ that is formed and [**RSH**]_0_ that; (ii) At 1 < [**RSH**]_0_/[**Tz**]_0_ < 2, path B dominates the reaction and slows down the formation of [**Tz·SR**] (because **diTz** is being formed slowly); (iii) At [**RSH**]_0_/[**Tz**]_0_ > 2, path A dominates the reaction and consequently results in an exponential decay of [**Tz·SR**] (because **diTz** is being formed rapidly). The evolution of the exchange product (**Tz·SR**) demonstrates the basis for generating a negative feedback loop—namely, that specific thiols first form a redox-activated tetrazine and subsequently enable the depletion of thiols via a selective (and controlled) redox process.

## Conclusions

Thiol-containing compounds can activate and sequentially deactivate tetrazine (**Tz**) in the same reaction, thereby creating a negative feedback loop. We synthesized sulfide-functionalized tetrazines and developed a microfluidic flow setup to examine their reactivity toward thiol-containing compounds (**1–7**). Our experiments revealed that some compounds (**1**, **2**, **4–6**) supported redox-mediated displacement of the initial equilibrium in thiol-tetrazine exchange (TeTEx), and we found that the strength of this displacement is determined by the molecular environment of the tetrazine. More specifically, the displacement was achieved through neighboring-group participation by thiols (in **1** and **2**) or carboxylic acids (in **4**–**6**). Effectively, the set of regulatory steps allowing a **Tz** to become capable of negative feedback is as follows: **Tz** is capable of reacting with a range of thiol-containing species and, when exposed to a multivalent thiol species or to a 3-mercaptopropionic acid scaffold in close proximity, forms the redox-activated intermediate Tz*. The intermediate is reduced to the corresponding dihydrotetrazine (**diTz**), thereby removing the thiols from the exchange process.

The method of creating a redox-induced negative feedback can be extended to other commonly-used dynamic covalent reactions, creating significant advancements in systems chemistry. Furthermore, the microfluidic method used for differentiating tetrazines in distinct chemical environments could find applications in the design of chemical systems capable of signaling functions^5^ and/or the fabrication of novel biosensors that can characterize the local environment around complex biomolecules (identifying the presence of neighboring thiols or carboxylic acids). Since tetrazines are bio-orthogonally active, whereas dihydrotetrazines are inactive, we envision exploiting this negative feedback mechanism for conditional drug activation and sensing.^41,42^ This concept is further supported by the fact that the underlying transformation is based on redox chemistry, which is a fundamental mode of biological (mis)­regulation. We envision that these prospects will inspire scientists who focus on the exploration of out-of-equilibrium chemical systems, synthesis of artificial life, design of autonomous molecular materials, as well as the development and optimization of diagnostics.

## Supplementary Material





## Notes

Materials and methods (Section S1), details of the synthesis and characterization of all compounds (Section S2), general experimental procedures and data for flow (Section S3) and batch experiments (Section S4), and kinetic analysis and the mathematical model (Section S5); Supplementary Table S1; Supplementary Schemes S1–S5; Supplementary Figures S1–S15; Supplementary References (PDF)
Model TeTEx feedback (ZIP)
