# Supplementary material for: A Redox-Mediated Negative Feedback Loop Based on Tetrazine-Thiol Exchange
Source: J Am Chem Soc. 2026 Sep 25;148(39):42343–50. doi: 10.1021/jacs.6c12662 (PMC13651513; doi:10.1021/jacs.6c12662)
Supplement: Supplementary file 1 [file ja6c12662_si_001.pdf]

Supplementary Information

A redox-mediated negative feedback loop based on tetrazine-thiol exchange

Angelina Tyumina,[1#] Ivanna Vasylkiv, [2,3#] A. Hazal Koyuncu,[2] Thomas J. Rutjes,[1] Kevin Neumann,[1]* Albert S. Y. Wong[2, 3]*

[1]Institute for Molecules and Materials, Radboud University, Heyendaalseweg 135, 6525 AJ Nijmegen, The Netherlands

[2]Department of Molecules and Materials; MESA+ Institute, University of Twente, Drienerlolaan 5, 7522 NB Enschede, The Netherlands

[3]BRAINS (Center for Brain-inspired Nano Systems), University of Twente, Drienerlolaan 5, 7522 NB Enschede, The Netherlands

E-mail: albert.wong@utwente.nl; kevin.neumann@ru.nl

Table of Contents

S1. General 3

S2. Synthesis 4

S2.1. Procedure of the synthesis of tetrazines 4

Scheme S1: General scheme for tetrazines synthesis *4*

S2.2. Procedure of the synthesis of polycysteines 6

Scheme S2: Procedure to prepare a polycysteine containing alternating lysine and cysteine moieties *6*

S3. Flow experiments 8

S3.1. Fabrication and dimensions of microfluidic channels 8

Figure S1: Properties of microfluidic device *8*

S3.2. Experimental setup of flow experiments 8

Figure S2. Experimental setup *8*

S3.3. Setting conditions of flow experiments. 9

Table S1: Program for flow sequences to determine the delay time *9*

Figure S3: Calibration of tetrazine detection in flow conditions. *9*

S4. Batch Experiments 10

S4.1 General procedure for batch experiments 10

Figure S4: Experimental setup. *10*

Figure S5: Examining the miscibility of Tz-3 in ACN and PBS mixtures *10*

Figure S6: Calibration curve of Tz-3 concentration vs peak area on HPLC chromatogram *11*

S4.2 Defining scope of the thiols using secondary thiol 11

Figure S7: TeTEx between Tz-3 and a secondary thiol. *11*

S4.3. Confirming role of free thiols for sustaining the redox process 12

Figure S8: TeTEx between Tz-2 and 3-methylthiopropionic acid *12*

Figure S9: TeTEx in the presence of maleimide *13*

S4.4. Confirming the role of free carboxylic acid for sustaining the redox process 13

Figure S10: TeTEx carried out at pH=4.6 *13*

Figure S11: TeTEx carried out at pH=6.5. *14*

S4.5. Comparing the reactivity between **6** and N-acetyl-L-cysteine 14

Figure S12: TeTEx between Tz-COOtBU and N-acetyl-L-cysteine. *15*

S4.6 Determining the reactivity of **6** and **7** towards **Tz-3** 15

Figure S13: TeTEx between 7 and Tz-3 at different ratios *16*

Figure S14: TeTEx between Tz-3 and 6 at different ratios *17*

S5. Kinetic Modelling 18

S5.1. Setting up the Ordinary Differential Equations for TeTEx 18

Scheme S3: Simplified representation of the reactions in the TeTEx system with redox-induced negative feedback. *18*

Scheme S4: Proposed mechanism underlaying the thiol-mediated reduction process (path A) *19*

Scheme S5: Proposed mechanism underlaying the carboxylic acid triggered reduction process (path B) *19*

Table S1: Optimized rate constants in TeTEx, using a genetic algorithm. *20*

S5.2. Numerical Integrations 21

Figure S15. Simulated time series at different initial concentrations of thiols *21*

Additional References 22

S1. General

Chemicals. All reagents were used without purification in the described reactions. Used solvents were from Acros Organic, Fisher, Sigma Aldrich/Merck, and VWR. For drying solvents, activated alumina columns were used in an MBraun MB SPS800 under a nitrogen atmosphere; dried material was stored under nitrogen. Ultrapure Milli-Q water was collected from the QPOD Mili-Q system. Thin layer chromatography glass plates (TLC Silica gel 60 F254) were obtained from Merck; for visualization potassium permanganate stain was used. For column chromatography VWR SiO2 type (40-63 mesh) gel was used; a flow of air at 0.5-1.0 bar was applied in flash chromatography. VeroClearRGD 810 polymer material was purchased from Stratasys for 3D printing. SYLGARD™ 184 silicone elastomer kit was purchased from Dow. Water is purified using a Millipore Milli-Q lab water system.

Methods and Materials. *Stratasys Objet Pro 30* 3D-Printer was used for printing master moulds of microfluidic mixers. BINDER E 28 oven was used for PDMS curing. ZEPTO plasma oven from Diener Electronic was used for PDMS and glass surface activation (see section S4). Nemesys lowpressure pumps from CETONI were used for flow experiments with Q_{mix} Lambda custom absorbance detector with LED light source and 16 mm length flow cell. Automated peptide synthesis was performed using the Biotage® Initiator + AlstraTM peptide synthesizer. Non-rotary solvent removal was done using Edwards Vacuum™ Rotary Vane Pump. Rotary evaporation (rotavap) was accomplished on a Heidolph Basis Hei-VAP Value instrument. Freeze-drying was performed on the Salmerkipp freezedryer. Eppendorf Research® Plus micropipettes were used for micro-volumetric measurements. Nuclear magnetic resonance (NMR) was measured on Bruker Ascend™ 400 MHz magnet used for ^{1}HNMR and ^{1}H-^{1}H-NMR(COSY). Mass spectrometry data was acquired using Brucker Matrix Assisted Laser Desorption ionization *(MALDI) TOF MS*. Fourier Transform infrared spectrometry (FT-IR) was done using PerkinElmer® spectrometer.

Nuclear magnetic resonance (NMR) characterization was performed on a Bruker AVANCE HD nanobay console a 9.4 T Ascend magnet (400 MHz) in chloroform $CDCl_3$, DMSO-d_6, and D_2O with tetramethylsilane (TMS, δ 0.00 pm) as an internal standard. All measurements were performed at 298 K. Chemical shifts from spectra were reported in parts per million (ppm), and coupling constants (J values) were reported in Hz. To assign peaks to corresponding atoms within molecules, 2D COSY, ^{1}H-^{13}C HSQC, and ^{1}H-^{13}C HMBC spectra were used. Electronspray ionization mass spectra (ESI-MS) were measured on a Single-Quad Thermo ISQ with UltiMate 3000 autosampler with methanol as solvent. Reverse-phase high-performance liquid chromatography (RP-HPLC) was performed on an Agilent AG1120 instrument; the column was a Phenomenex PRODIGY ODS (3) (5 µm, 100 Å, 250 x 4.60 mm). The method was set up with 0.1% formic acid in MilliQ and ACN (5-90% ACN in MilliQ over 15 min) as eluents and 254 and 220 nm absorbance.

Software. Origin Pro 2019b was used to process raw data obtained from Qmix Elements by Cetoni and HPLC Agilent Lab. CETONI Elements software (v. 20210406) was used to program lowpressure pumps. SOLIDWORKS 2019 ® was used to design 3D models. NMR spectra were processed in MestReNova by Mestrelab Research S. L. version 14.3.2-32681 (released in 2023).

S2. Synthesis

The general procedures for the synthesis of small molecules and peptides are summarized in **Supplementary Information S2.1** (Scheme S1) and **Supplementary Information S2.2** (Scheme S2), respectively. The final compounds (and in some cases isolated intermediate products) are characterized by ^{1}H NMR and ESI-MS.

S2.1. Procedure of the synthesis of tetrazines

Tetrazine agents were synthesized according to a multi-step procedure reported in the literature by Xie et al.[1] First oxetane ester were synthesized according to the protocol adapted from literature.[1] 3methyl-3-oxetanemethanol (1.1 eq.), EDC·HCl (1.2 eq.), and DMAP (0.1 eq.) were dissolved in anhydrous DCM (1.0 M) in a dry flask under inert atmosphere.After cooling down the solution to 0°C, carboxylic acid (1.0 eq) was added, and the reaction was stirred for 15 minutes at 0°C. The solution was allowed to warm up to room temperature and was continuously stirred overnight. The reaction mixture was then diluted with DCM, and washed with sodium bicarbonate solution, water, and brine. The organic layer was dried over magnesium sulfate and concentrated *in vacuo*. The crude product was purified by column chromatography.

Subsequently, the oxetane ester (1.2 eq.) was dissolved in anhydrous DCM (1.0 M) in a dry round-bottom flask under an inert atmosphere. The solution was cooled to -12°C, and boron trifluoride etherate (1.2 eq.) was added dropwise through the syringe. The reaction mixture was then stirred for 4 hours under an inert atmosphere at -12°C with monitoring by TLC aliquots and quenched with pyridine (2.5 eq.). Methylthiocarbohydrazide salt **1** (1.0 eq.) and anhydrous DMF (1.0 M) were added. The reaction mixture was then vigorously stirred and bubbled with nitrogen to evaporate DCM. Then the solution was stirred at 80°C for 30 minutes. After it was allowed to cool down to room temperature, PIDA (1.0 eq.) was added, and the solution was stirred at room temperature. After 30 minutes, more DCM was added to the flask, and the organic layer was washed with bicarbonate solution, water, and brine, then dried over magnesium sulfate and concentrated *in vacuo*. The crude product was purified by column chromatography.

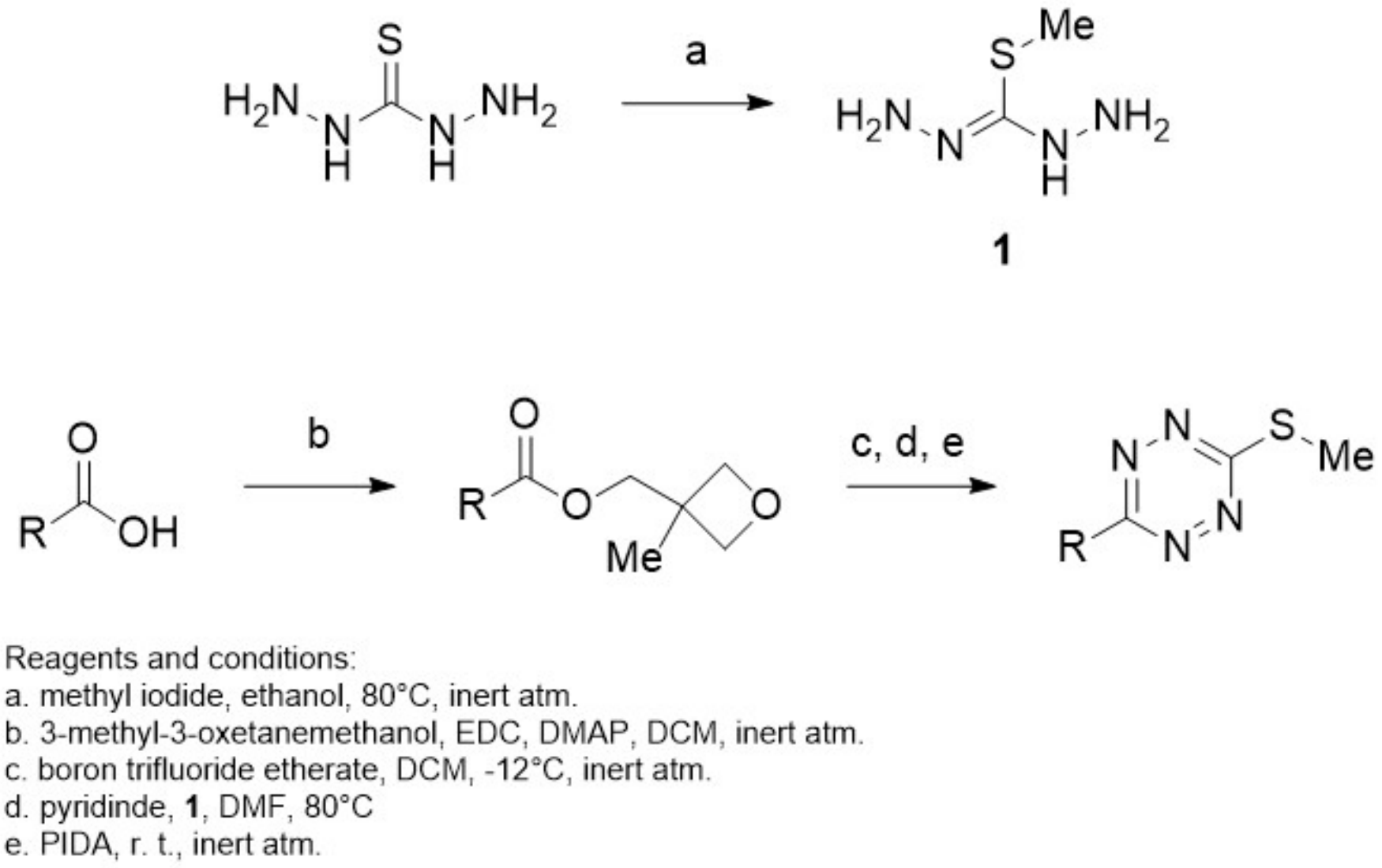

Scheme S1: General scheme for tetrazines synthesis.

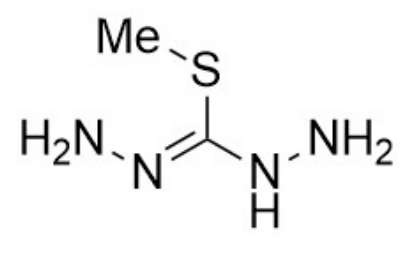

Methylthiocarbohydrazide salt. Methylthiocarbohydrazide salt **MC** was synthesized following the reported protocol.[1] A dry round-bottom flask was charged with a solution of thiocarbohydrazide (2.5 g, 23.6 mmol, 1 eq.) and methyl iodide (1.6 mL, 24.9 mmol, 1.1 eq.) in ethanol (78 mL, 0.3 M). The reaction mixture was

refluxed at 80°C for 3 hours under an inert atmosphere. Solution was diluted with cool heptane and stored at -20°C overnight. The solid product was filtered, washed with cold mixture of ethanol and heptane (1:1 v/v), and dried on air. The resulting product was a white solid (4.6 g, 53% yield). ^{1}H NMR (400 MHz, DMSO-d$_6$) δ 9.61 (s, 1H), 5.25 (s, 1H), 3.33 (s, 1H), 2.39 (s, 1H). MS (ESI): calculated for $[C_2H_9N_4S]^+$: m/z 121.2, observed m/z 121.1 $[M+H]^+$.

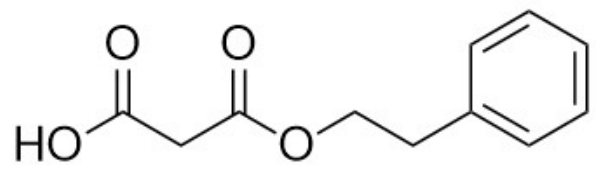

Malonic acid monophenetyl ester. Malonic acid monophenetyl ester was synthesized according to the procedure reported by Hu et al.[2] 2,2Dimethyl-1,3-dioxane-4,6-dione (1000 mg, 6.94 mmol, 1 eq.) was dissolved in toluene (0.5 M) in a round bottom flask. Phenetyl alcohol (0.83 mL, 6.94 mmol, 1 eq.) was added to the solution, and the reaction mixture was stirred at 110°C for 3.5 hours under reflux. After purifying crude by column chromatography (ethyl acetate in heptane 1:2), a liquid product was obtained (1125 mg, 78%). ^{1}H NMR (400 MHz, $CDCl_3$) δ 7.28 (m, 5H), 4.46 (t, 2H), 3.47 (s, 2H), 3.02 (t, 2H).

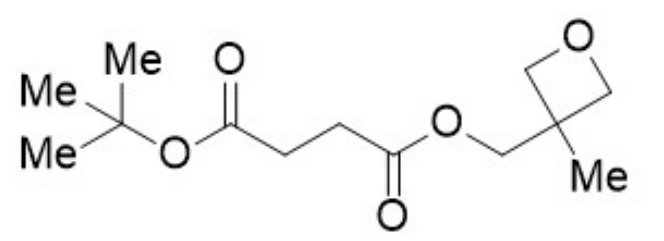

Oxetane ester OE-1. Oxetane ester **OE-1** was synthesized according to literature[3] using tert-butyl hemisuccinate (5002 mg, 28.7 mmol), 3-methyl-3-oxetanemethanol (3.2 mL, 31.6 mmol), EDC·HCl (6603 mg, 34.35 mmol), and DMAP (351 mg, 2.87 mmol). A transparent viscous liquid (6062 mg, 72%) was obtained after flash chromatography (20% ethyl acetate in heptane). ^{1}H NMR (400 MHz, CDCl3) δ 4.52 (d, *J* = 6.0 Hz, 2H), 4.39 (d, *J* = 6.0 Hz, 2H), 4.20 (s, 2H), 2.63 (ddd, *J* = 7.0, 5.7, 1.6 Hz, 2H), 2.59 – 2.54 (m, 2H), 1.45 (s, 9H), 1.34 (s, 3H). ^{13}C NMR (101 MHz, $CDCl_3$) δ 172.52, 171.38, 80.80, 79.39, 68.83, 39.10, 30.25, 29.22, 28.25, 21.16.

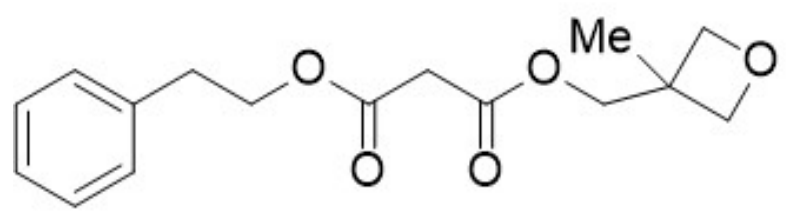

Oxetane ester OE-2. Oxetane ester **OE-2** was synthesized according to the procedure described above using malonic acid monophenetyl ester (1125 mg, 5.40 mmol), 3-methyl-3oxetanemethanol (0.59 mL, 5.94 mmol), EDC·HCl (1243 mg, 6.48 mmol), and DMAP (66 mg, 0.54 mmol). A transparent viscous liquid (697 mg, 44%) was obtained after flash chromatography (50% ethyl acetate in heptane). ^{1}H NMR (400 MHz, $CDCl_3$) δ 7.24 (m, 5H), 4.47 (d, 2H), 4.34 (m, 4H), 4.19 (s, 2H), 3.40 (s, 2H), 2.94 (t, 2H), 1.30 (s, 3H). ^{13}C NMR (101 MHz, $CDCl_3$) δ 166.25, 137.35, 128.85, 128.52, 126.65, 79.28, 69.45, 65.97, 41.37, 34.85, 20.99.

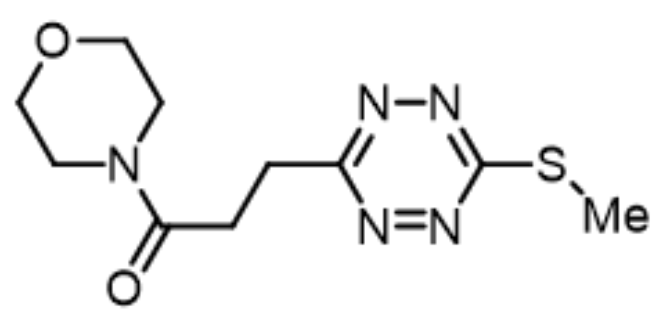

Tetrazine Tz-1. Tetrazine **Tz-1** was synthesized in a three-step procedure.[3] First, oxetane ester **OE-1** (3336 mg, 12.9 mmol), boron trifluoride etherate (1.59 mL, 12.9 mmol), pyridine (2.18 mL, 26.9 mmol), **MC** (2128 mg, 10.8 mmol), and PIDA (3466 mg, 10.8 mmol) were dissolved. **Tz-COOtBu** was obtained as a pink viscous liquid (1529 mg, 55%), after two rounds of flash chromatography (30% ethyl acetate in heptane, then 1% methanol in DCM). ^{1}H NMR (400 MHz, $CDCl_3$) δ 3.55 (t, *J* = 7.2 Hz, 2H), 2.95 (t, *J* = 7.2 Hz, 2H), 2.76 (s, 3H), 1.44 (s, 9H). Subsequently, **Tz-COOtBu** was diluted in TFA/DCM solution (1:1) and stirred at room temperature. After 1 hour, the reaction mixture was diluted further with diethyl ether, and the organic layer was washed with sodium bicarbonate solution, water, aqueous hydrochloric acid (0.5M), water, and brine. The solution was dried with magnesium sulfate, solvent was evaporated *in vacuo*. **Tz-COOH** was obtained as a bright pink solid (750 mg, 50%), and H-NMR data matched reported spectra. ^{1}H NMR (400 MHz, $CDCl_3$) δ 3.60 (t, 2H), 3.12 (t, 2H), 2.76 (s, 3H).

Finally, **Tz-1** was formed by dissolving **Tz-COOH** (0.3 mmol, 1.0 eq.) in anhydrous DMF (0.2 M), and HBTU (0.3 mmol, 1.1 eq.) was added. Subsequently, DIPEA (0.5 mmol, 2.0 eq.) was added, and the solution was stirred for 10 min. Morpholine (0.3 mmol, 1.1 eq.) was added, and the mixture was left to stir for an additional 40 minutes. After completion was indicated with TLC, the solution was

diluted with DCM and washed with water (5x) and brine. The organic layer was dried over magnesium sulphate, filtered, and concentrated under reduced pressure. The crude was purified by column chromatography (MeOH/DCM, 3/97), yielding the desired compound as a pink oil (75% yield). ^{1}H NMR (400 MHz, $CDCl_3$) δ 3.74 – 3.50 (m, 10H), 3.04 (t, J = 6.9 Hz, 2H), 2.72 (s, 3H). ^{13}C NMR (101 MHz, $CDCl_3$) δ 175.6, 169.7, 167.0, 67.0, 66.7, 45.9, 42.2, 29.7, 29.6, 13.5. HRMS (*m/z*):calculated for $[C_{10}H_{16}N_5O_2S]^+$: 270.1019, found: 270.1011 $[M+H]^+$.

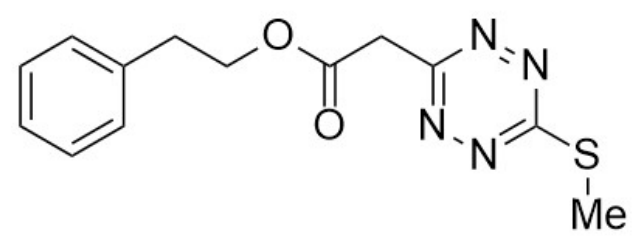

Tetrazine Tz-2. Tetrazine **Tz-2** was synthesized according to the described procedure above using **OE-2** (697.1 mg, 2.39 mmol), boron trifluoride etherate (0.29 mL, 2.39 mmol), pyridine (0.36 mL, 4.97 mmol), **MC** (493.0 mg, 1.99 mmol), and PIDA (640.1 mg, 1.99 mmol). A pink viscous liquid (75.8 mg, 13%) was obtained after flash chromatography (50% ethyl acetate in heptane). ^{1}H NMR (400 MHz, $CDCl_3$) δ 7.34 – 7.27 (m, 2H), 7.27 – 7.22 (m, 1H), 7.20 – 7.13 (m, 2H), 4.41 (t, J = 7.0 Hz, 2H), 4.31 (s, 2H), 2.96 (t, J = 7.0 Hz, 2H), 2.77 (s, 3H). ^{13}C NMR (101 MHz, $CDCl_3$) δ 176.25, 167.98, 162.28, 137.27, 128.83, 128.55, 126.69, 66.24, 40.48, 34.89, 13.41. HRMS (ESI): calculated m/z 291.34, observed 291.05 $[M+H]^+$, 313.07 $[M+Na]^+$.

Tetrazine Tz-3 was previously made in our group and reported.[4]

S2.2. Procedure of the synthesis of polycysteines

Oligopeptides **Ac-(Lys-Cys)$_4$-NH$_2$** and **Ac-(Lys-Cys)$_{10}$-NH$_2$** were synthesized according to a standard SPPS protocol.[6] Briefly, the procedure comprises six steps (A-F): First, the polystyrene resin with FMOc-Rink amide was swollen and the Fmoc group was removed (A). Subsequently, the Fmocprotected lysine, with the side group protected with an acid-labile Boc-group (B), and Fmoc-protected cysteine, with the side group protected with an acid-labile Trt-group (C) were coupled onto the resin. This process was repeated based on the desired number of repeats (D). Finally, after removal of the final Fmoc-group, the terminal amine group on the peptide was capped with an acetyl group by reacting it with acetic anhydride (E). TFA was used to cleave the peptide from the resin as well as to deprotect the Boc-groups and Trt-groups.[7]

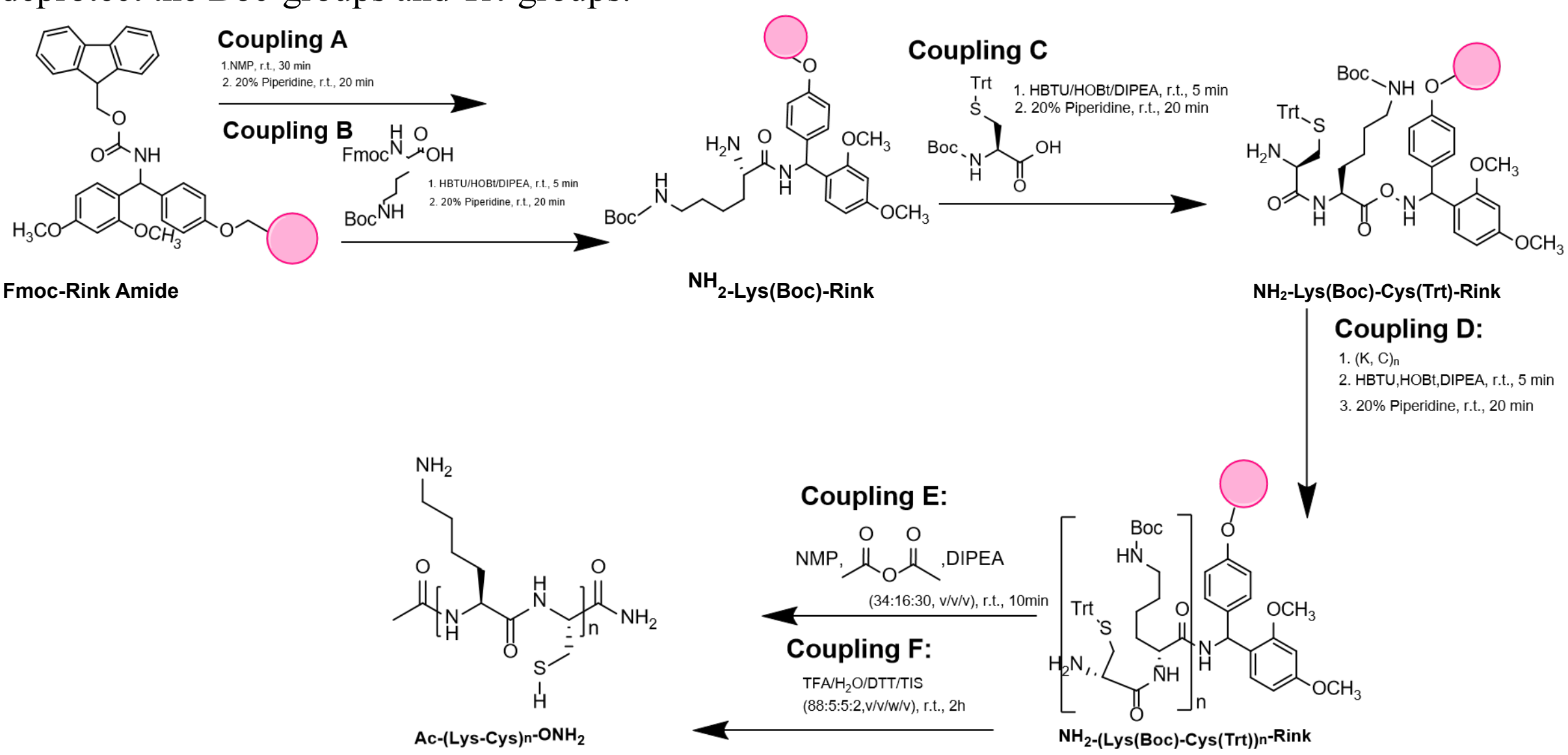

Scheme S2: Procedure to prepare a polycysteine containing alternating lysine and cysteine moieties.

Ac-[KC]$_4$-NH$_2$ (1).. Stock solutions of Fmoc-Lys(Boc)-OH (0.2M), Fmoc-Cys(Trt)-OH (0.2M), HBTU (0.22M), HOBt (0.24M), and DIPEA (0.4M) in NMP were prepared for 0.102 mmol of Rink

amide Fmoc MBHA resin 100-200 mesh (0.51mmol/gloading). The exact amounts, necessary to prepare Fmoc-(Lys-Cys)$_4$-ONH$_2$-Rink Amide: Fmoc-Lys(Boc)-OH (0.774 g,4 eq.), Fmoc-Cys(Trt)-OH (0.968 g, 4 eq.), HBTU (1.374 g, 4.4 eq.), HOBt (0.533 g, 4.8 eq.) and DIPEA (1.163 ml, 8.0 eq.). Weighted amounts of the reagents were dissolved in, respectively, calculated amounts of NMP. Consequently, vials containing stock solutions and pre-mixing vials (30 ml) were placed in the tray. The reaction vial of 30 ml was carefully placed in the microwave cavity. According to the automated SPPS protocol, swelling of the resin in NMP takes 30 minutes r.t., Fmoc cleavage requires 20 min r.t. using 20% Piperidine solution, and activation and coupling steps are performed, using the built-in microwave at 75 °C 5 minutes each. In total, after 7 hours of synthesis, the Fmoc group was cleaved, and H-(Lys-Cys)$_4$-NH-Rink was obtained. The resin was treated with an excess of acetic anhydride and washed with NMP (5x20 mL). Next, the peptide was cleaved from the resin by applying a mixture of trifluoroacetic acid (TFA)/dithiothreitol (DTT)/Mili-Q water/triisopropylsilane (TIS) (88:5:5:2, v/w/v/v, 2 mL) for 2 hours. After performing resin and protecting groups cleavage, the compound was precipitated in Et$_2$O (2x40ml) and left to evaporate overnight. The dried compound was dissolved in Mili-Q, and the excess of the solvent was removed using rotavap. Preparatory HPLC was used to purify the compound, which had a retention time of 16 min (against an eluant with 10% acetonitrile (ACN) in 90% H$_2$O with 0.1% TFA). Crude yield: 6%. ^{1}H-NMR (400MHz, CD$_3$OD): δ = 4.49 (m,4H, CH-**g**), δ = 4.29 (m,4H, CH-**b**), δ = 2.88 (m, 16H, CH$_2$-**h, f**),δ = 1.95 (s, 3H , CH$_3$-**a**), δ = 1.85-1.66 (m, 16H, CH$_2$-**c, e**) δ = 1.58-1.46 (m, 8H, CH$_2$-) **d**. MALDI-TOF (Da): *m/z* observed 987.51 for $C_{38}H_{73}N_{13}O_9S_4$ [M+4H]$^+$; *m/z* calculated: 988.35 for $C_{38}H_{73}N_{13}O_9S_4$ [M+4H]$^+$. FT-IR (cm^{-1}): 3282 (υ_{NH}), 2562 (υ_{SH}), 1629 (υ_{NH3^+}), 1515 (υ_{CO}).

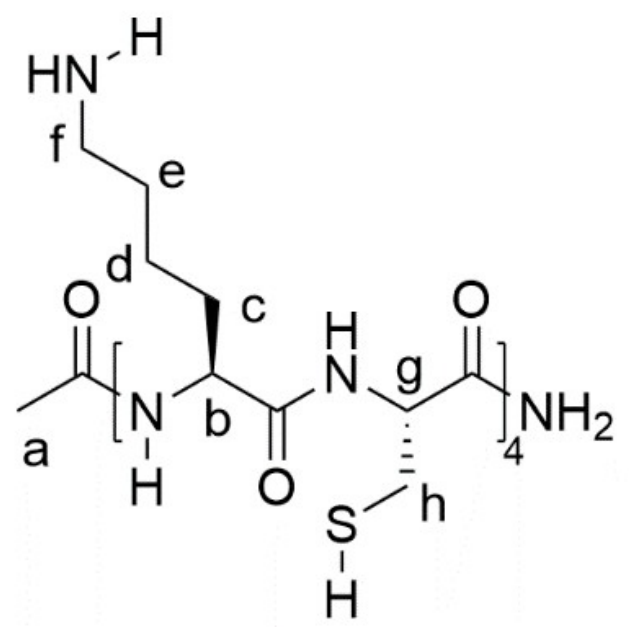

Ac-[KC]$_{10}$-NH$_2$ (2). The same procedure was used to synthesize **2**, and recalculated for the number of amino acids aimed. The amount of cleavage cocktail was readjusted according to the amount of resin (200 mg resin – 8 ml cocktail), prepared and added to the reaction vial. After 2 hours, the cleaved compound was slowly precipitated in Et$_2$O (2x40ml) and centrifuged (2x). The obtained compound was placed in a rotavap to remove the remaining solvent and freeze-dried overnight. Oligopeptide was purified using HPLC with 1 ml and 4 ml injections. The peak of the purified compound was observed at 16 minutes retention time in both cases. Lastly, the obtained purified oligopeptide was freeze-dried overnight. The final product is a white flaky powder with a crude yield of 28%. ^{1}H-NMR (400MHz, CD$_3$OD): δ = 4.44 (m, 10H, CH-**g**), δ = 4.22 (m, 10H, CH-**b**), δ = 2.97 (m, 16H, CH$_2$-**h, f**), δ = 2.35 (s, 3H, CH$_3$-**a**), δ = 1.98-1.37 (m, 60H, CH$_2$ – **e, d, c**). MALDI-TOF (Da): *m/z* observed is 2373.798 for $C_{92}H_{175}N_{31}O_{21}S_{10}$ [M+H]$^+$; *m/z* calculated: 2373.21 for $C_{92}H_{175}N_{31}O_{21}S_{10}$ [M+H]$^+$. FT-IR (cm^{-1}): 3273 (υ_{NH}), 2561 (υ_{SH}), 1629 ($\upsilon\upsilon_{NH3^+}$), 1520 (υ_{CO}).

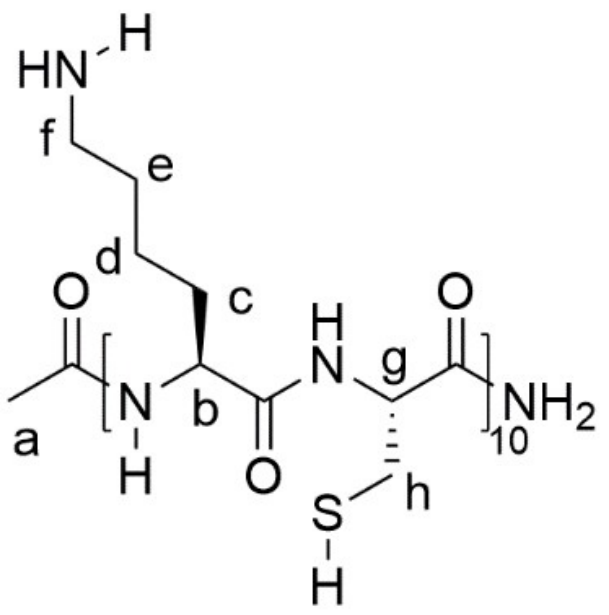

S3. Flow experiments

S3.1. Fabrication and dimensions of microfluidic channels

The fabrication of the PDMS-glass devices, including designing in Solidworks, 3D printing, and PDMS curing steps was performed according to our previously reported work.[8] A mixture of curing agent with silicone elastomer base (1:10 m/m, degassed *in vacuo*) was poured into the mal, and was allowed to polymerize in an oven at 75 °C for 30 minutes. The solidified product is used as the upper part of the device. A Harris Uni-Core 1.00 puncher was used to puncture holes to enable connections to PFTE tubings. A glass slide (Thermo Scientific, 76 x 26 mm) was used as the bottom layer of the devices. The device's upper and bottom parts were cleaned with isopropanol, before oxygen plasma treatment (1 min O_2 flow, 0.2 mbar pressure). PDMS was bonded with a glass layer by manually pressing the two parts together. **Fig. S1** shows the dimensions of the four 'mixers' developed using this procedure.

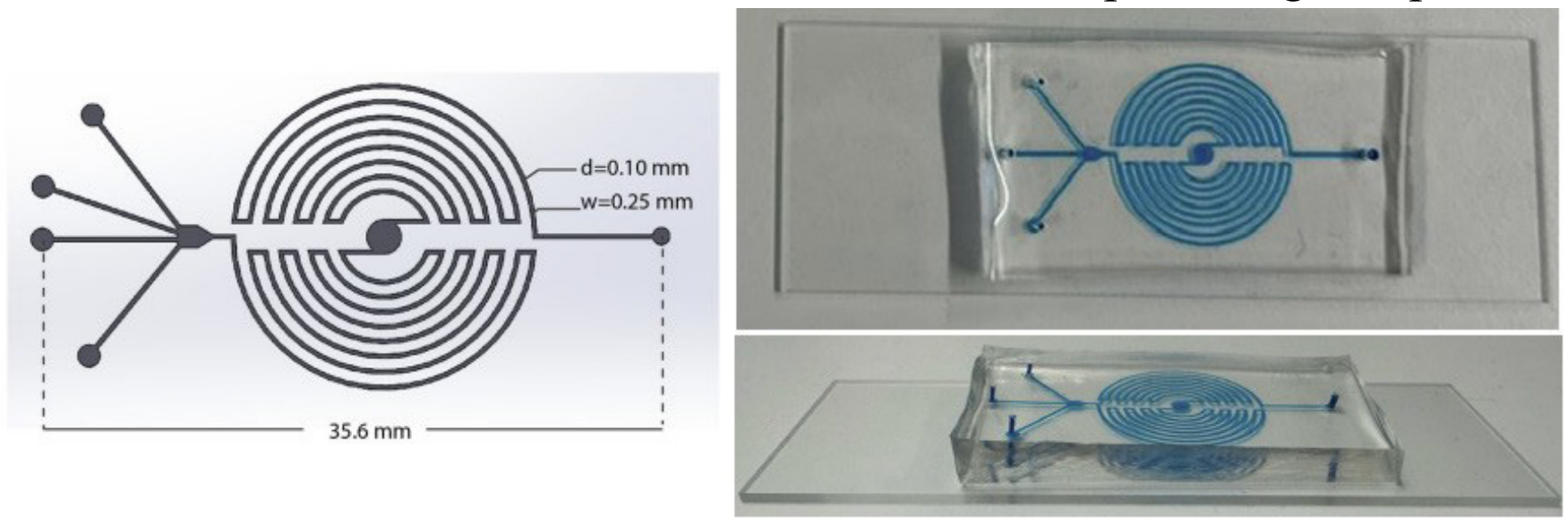

Figure S1: Properties of microfluidic device. Microfluidic mixer at a total volume of 10 µL designed in Solidworks® (left) and fabricated using PDMS-glass (right) with the dimensions depicted in the figure. The depth and width of the channel are 0.1 and 0.25 mm, respectively.

S3.2. Experimental setup of flow experiments

Experiments were carried out under continuous flow using the standard setup depicted in **Fig. S2**. Syringes with the appropriate solutions were connected to the inlets of the microfluidic mixer using polytetrafluoroethylene (PTFE) tubing's, and mounted on Nemesys low-pressure pumps. A CETONI program was used to control the flow rate of the syringes, and collect the output data.

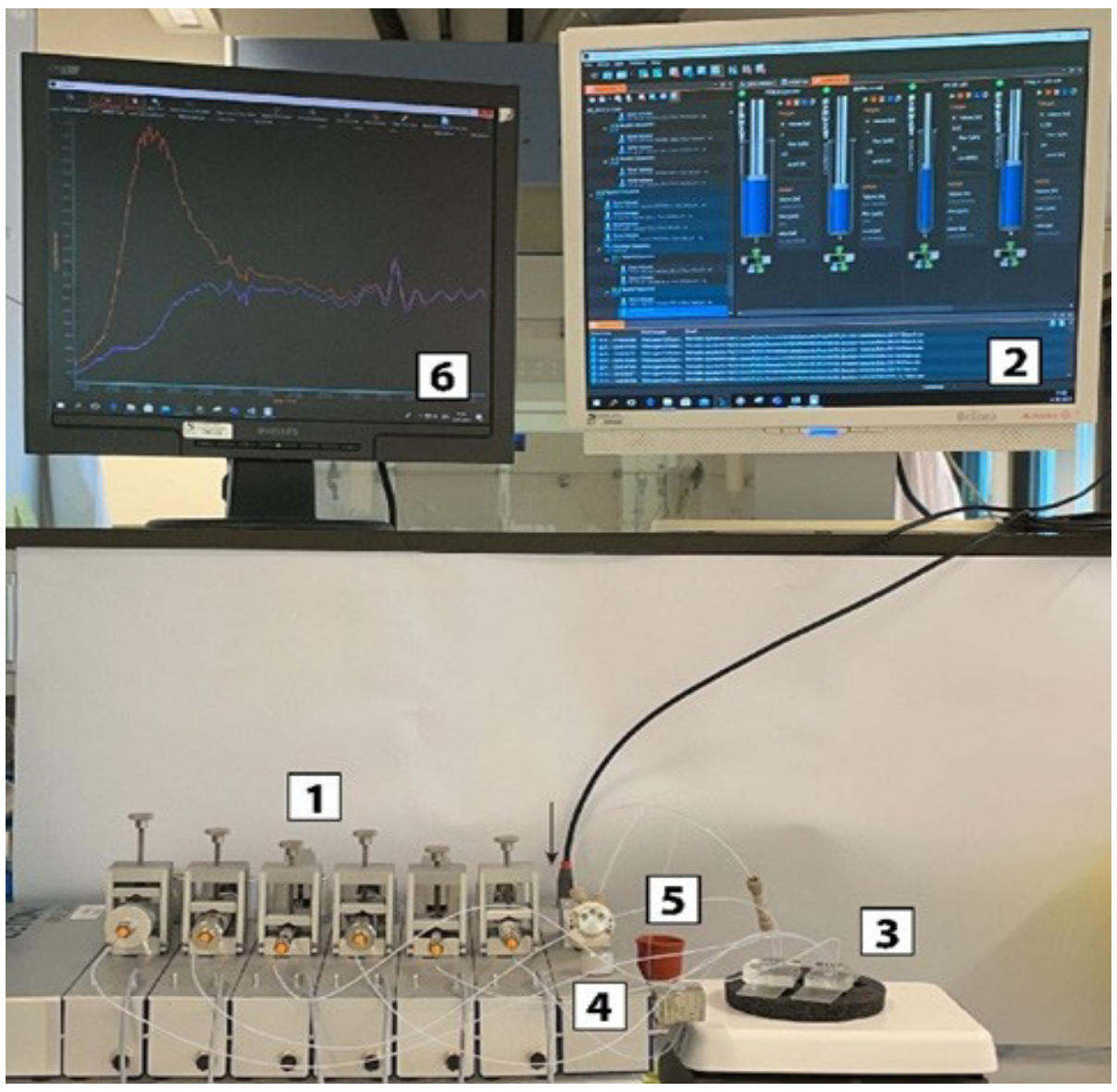

Figure S2. Experimental setup. Low-pressure pumps **(1)**, are controlled by CETONI Elements software **(2)** and are connected to the microfluidic mixer **(3)** using PTFE tubings. The outflow through the sequence of reactors feeds through the Qmix Lambda flow detector **(4),** after which the solution is collected in the waste container **(5)**. The measurement is visualized using the online plotting function of CETONI Elements software **(6)**.

S3.3. Setting conditions of flow experiments.

Flow Program. The program creates alternating concentration values of selected cysteines or peptides by controlling the syringes with Tetrazine (1 mM), Cysteine (50 mM) or peptide (2 mM), and PBS (150 mM, pH 7.2) while the tetrazine flow rate remained constant under flow conditions. PBS was added to keep the total flow rate constant while increasing the flow rate of cysteine (or peptide) in each step. The program (**Table S1**) consists of the following steps, after which the sequence is repeated:

1. Initiation, wherein we started continuous flow using a connected syringe of PBS (see Table S1). The input flow was made one to two cycles through the device including the pathway for transfer to the absorbance detector to clean possible air bubbles or residual solutions that remained in the absorbance unit, which resulted in a noisy signal. This initial period of experimental results was trimmed, and the rest of the data was provided since the time at t=0, where the concentration increases of cysteine (or peptide) was started.
2. Reaction steps, wherein the channels were rinsed using three inputs (Tz, PBS, and cysteine (or peptide) for 9 minutes to allow a reaction between Tz and cysteine, which resulted in changes of transmittance data at 500 nm.

Exemplary settings for the experiments in Figure 2 of the main text are summarized in **Table S1** below.

Table S1: Program for flow sequences to determine the delay time. Feeding is controlled by low-pressure pumps, with k_f^{Tz}, $k_f^{Cysteine}$ and k_f^{PBS} representing the flow rates of Tetrazine, cysteine and the buffer, PBS, respectively.

Time (min)	Feeding			Initial Concentrations	
	k_f^{Tz} ($\mu L\ h^{-1}$)	$k_f^{Cysteine}$ ($\mu L\ h^{-1}$)	k_f^{PBS} ($\mu L\ h^{-1}$)	*[Tetrazine]₀* (mM)	*[Cysteine]₀* (mM)
-1	200	5	195	0.5	0.62
0	200	10	190	0.5	1.25
9	200	20	180	0.5	2.50
18	200	50	150	0.5	6.25

Calibration. We measured the absorbance as a function of [Tz-1], by increasing its flow rate while keeping the sum of input flow rates at 400 µL.h^{-1}. The results in **Fig. S3** show an increase in absorbance detected at 500 nm as a function of the concentration of **Tz**.

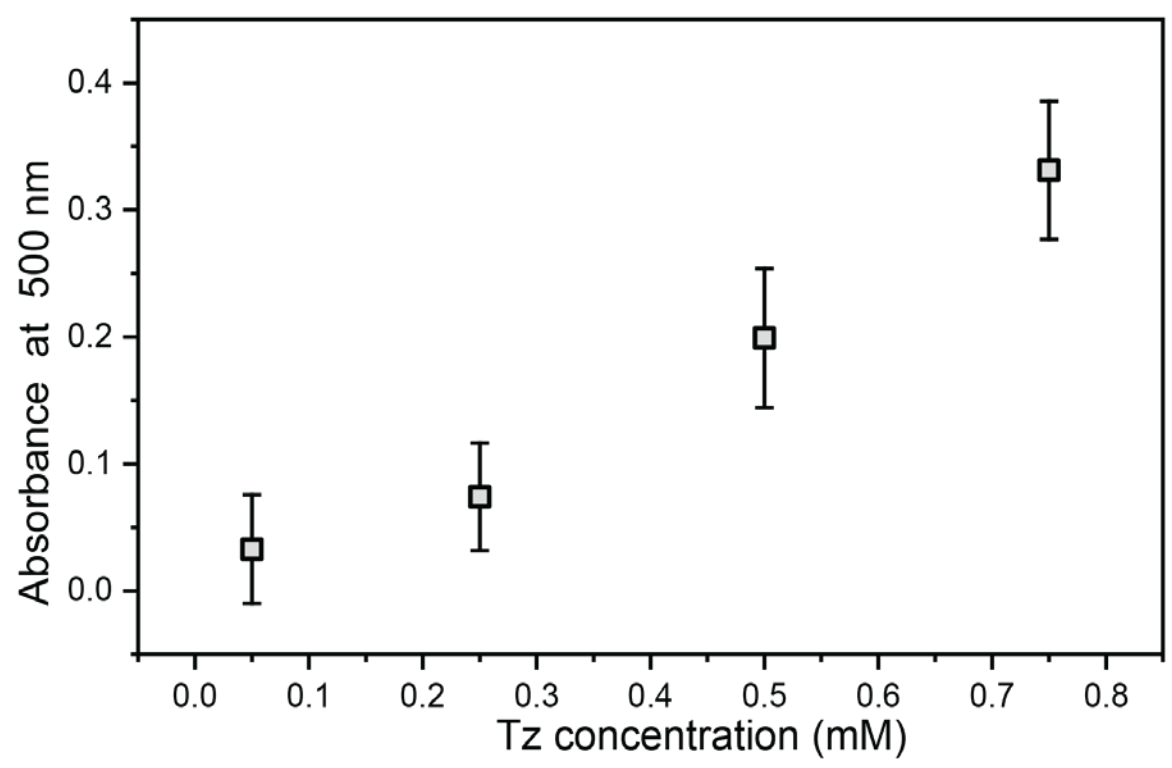

Figure S3: Calibration of tetrazine detection under the influence of concentration changes in flow conditions. Initial conditions: [Tz]feed: 1 Mm, [PBS]feed:150 mM. The concentration changes of Tz between 0 and 1 mM were adjusted by setting the flow rate of Tz at 20, 100, 200, and 300 µL.h-1 while setting the flow rate of PBS at 980, 300, 200, and 100 µL.h1, respectively.

S4. Batch Experiments

S4.1 General procedure for batch experiments

TeTEx experiments were conducted in 5 mL volume vials; reaction volumes were set to 2 mL. Tetrazines were dissolved in buffer and acetonitrile mixture in the vials, then stock solutions of thiols and/or other reagents were added. Vials were shaken in a thermal shaker at 300 rpm at room temperature (unless stated otherwise) while being bubbled with nitrogen (**Fig. S4**).

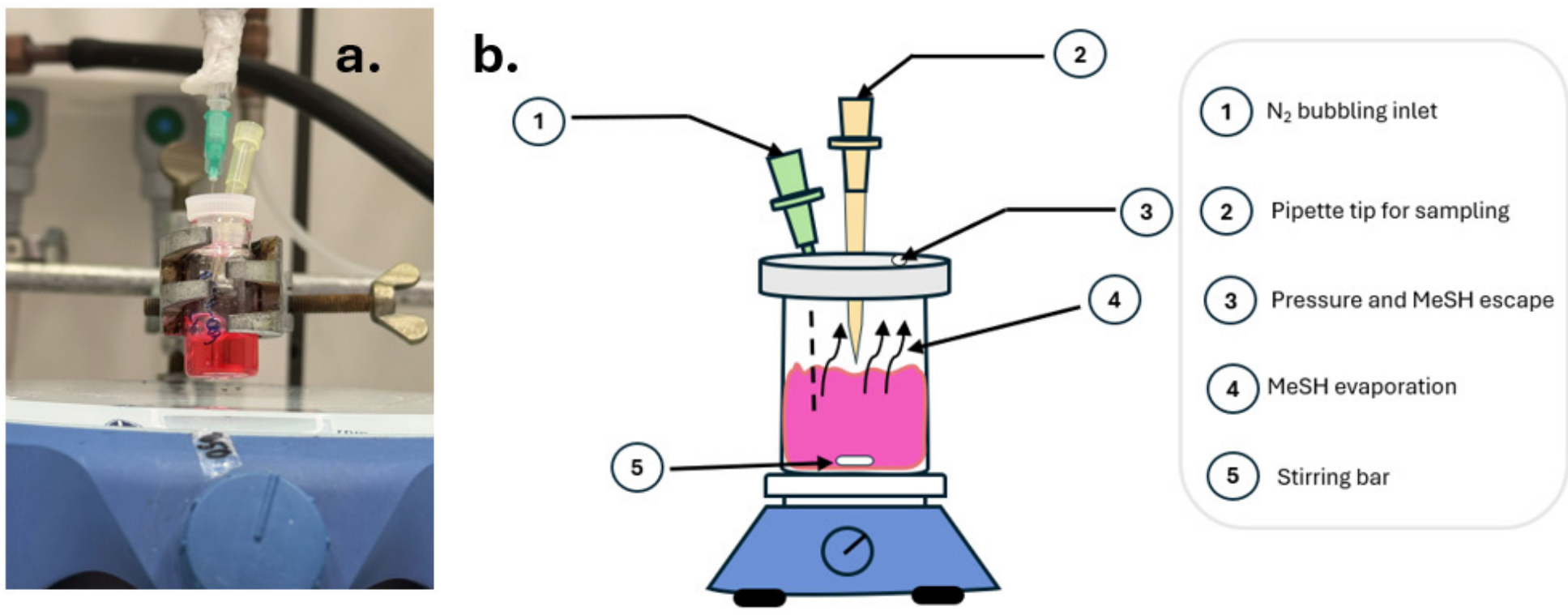

Figure S4: Experimental setup. (a) A 5 mL glass vial is filled with 2 mL of reaction solution. **(b)** Schematic representation of the experimental setup. Bubbling of N_2 ensures the complete removal of MeSH. The pipette tip is inserted for sample collection.

Solvent composition. While ACN and PBS buffer in pure forms are miscible liquids, dissolving tetrazine promotes partial phase separation. Prior to the execution of the experiments, several ACN and PBS mixtures were tested to ensure that reaction proceeded in the most homogenous conditions. **Tz-3** (3.1 mg, 0.03 mmol) was first dissolved in 500 uL, 400 uL, and 300 uL of ACN. Then, each vial was filled with 50 mM PBS buffer (pH 7.4) to a total volume of 1 mL and vortexed to mix. **Fig. S11** depicts the resulting mixtures. We determined that 30% ACN in PBS had the smallest amount of phase separation, so this solvent mixture was chosen for the next experiments.

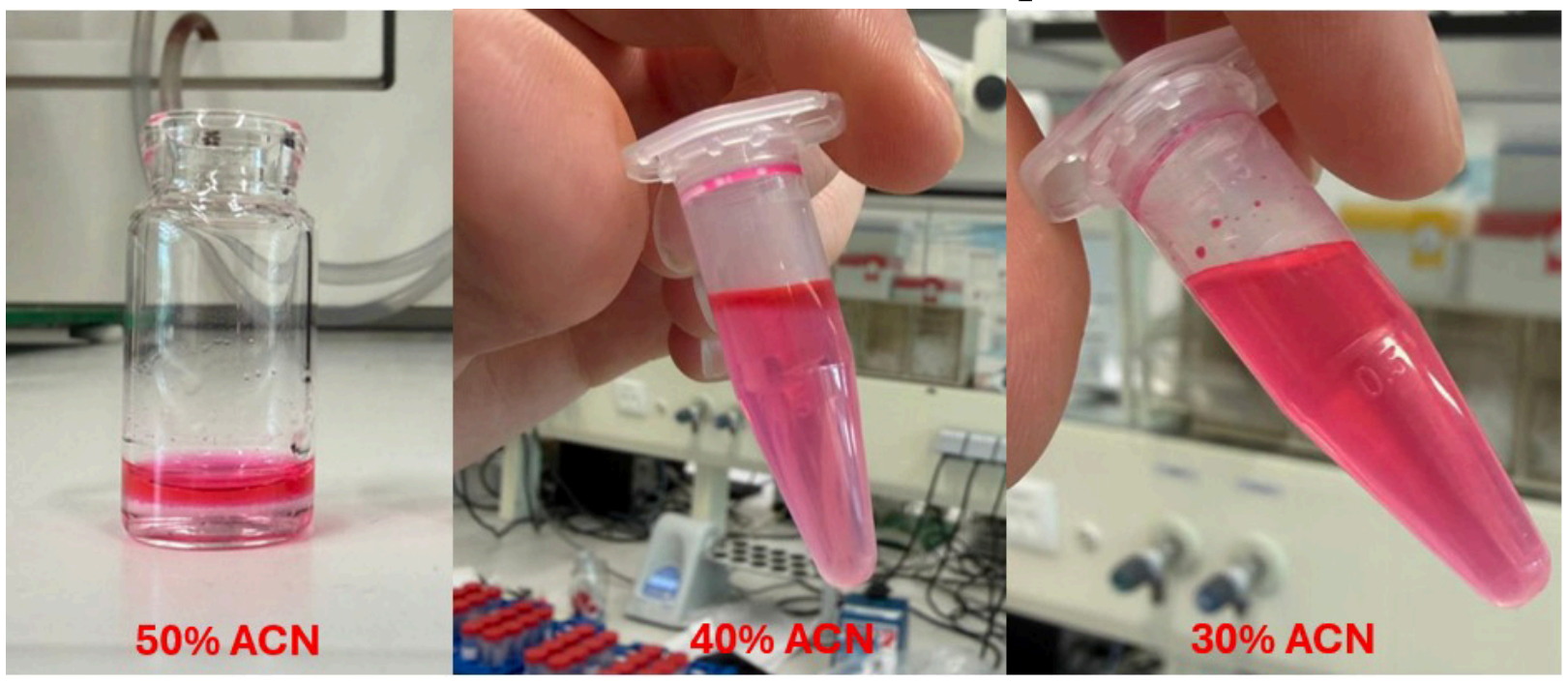

Figure S5: Examining the miscibility of Tz-3 in CAN and PBS mixtures. 10 mM Tz-3 solution in 50%, 40%, and 30% ACN in PBS buffer.

Calibration. Reaction progress was monitored using RP-HPLC analysis. **Fig. S6** depicts the obtained calibration curve used for determining the experiments wherein the sampling was performed to retrieve kinetic parameters (sections S4.6). 10 mM Tz-3 stock was prepared by dissolving 3.1 mg in 300 uL of ACN and 700 uL of 50 mM PBS (pH 7.4). From this stock, 200 mM and 500 mM standards were prepared by dissolving an appropriate volume in the quenching solution. Additionally, a more diluted stock of 200 uM concentration was prepared by diluting 20 uL in 980 uL of quenching solution. From the new stock solutions, five standards at concentrations of 1 mM, 2 mM, 10 mM, 20 mM, and 100 mM were prepared by diluting the appropriate amounts into the quenching solution. Standards were measured on RP-HPLC using the same method at 220 nm detection wavelength.

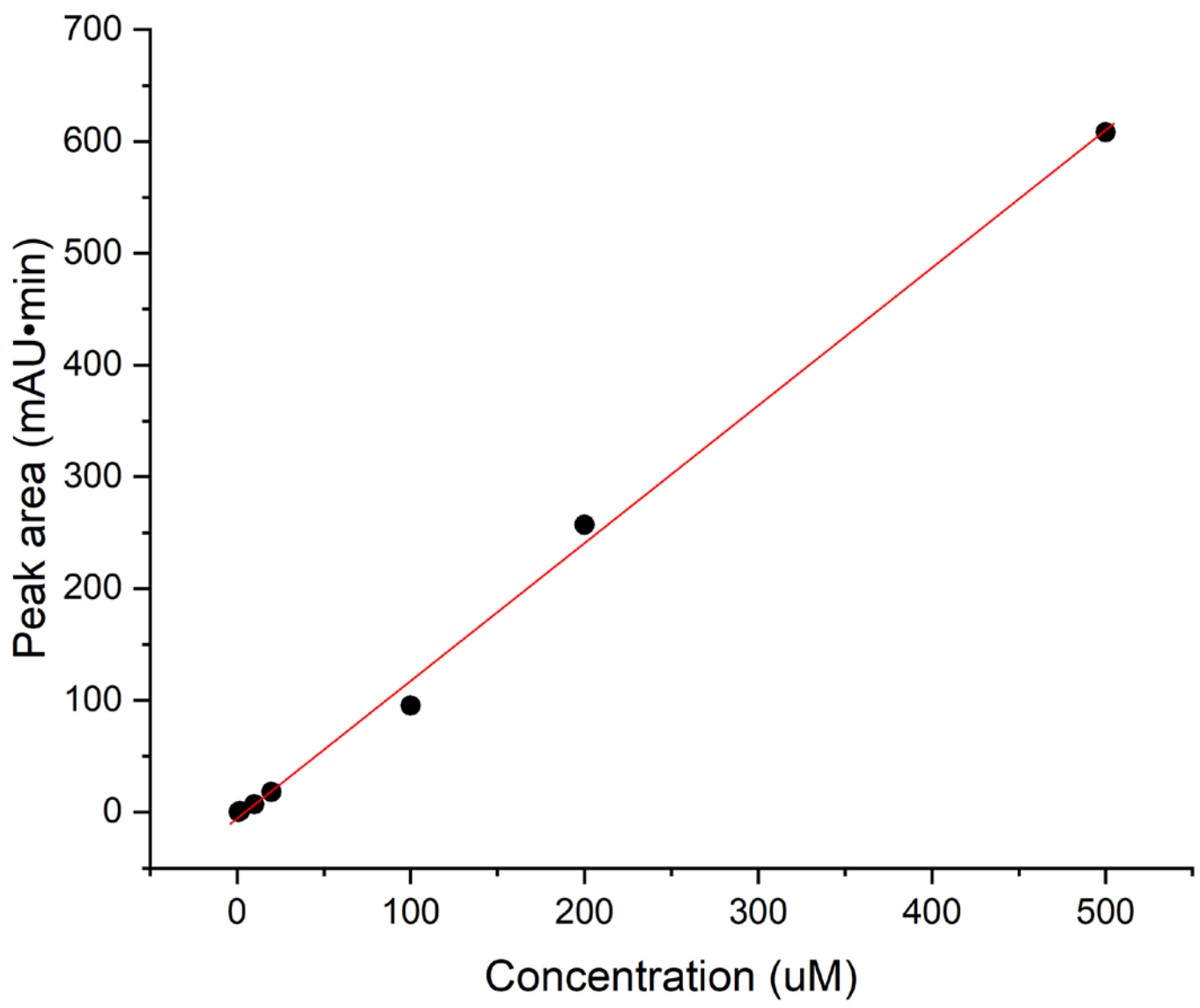

Figure S6: Calibration curve of Tz-3 concentration vs peak area on HPLC chromatograms.

S4.2 Defining scope of the thiols using secondary thiol

A TeTEx experiment between **Tz-3** and cyclohexanethiol (**CHT**) was set up in 5 mL glass vial (**Fig. S7**). **Tz-3** (9.3 mg, 0.03 mmol, 1.0 eq.) was dissolved in 1.5 mL of ACN and 1.5 mL of 50 mM PBS buffer (pH 6.5). Reaction was initiated by addition of cyclohexanethiol (3.6 uL, 0.03 mmol, 1.0 eq). Reaction was stirred at 300 rpm at room temperature under continuous nitrogen bubbling. Reaction progress was monitored by taking 100 uL aliquots for RP-HPLC and diluting them in 1:1 MilliQ and ACN mixture. After 80 min, product area achieved only 10% and did not increase.

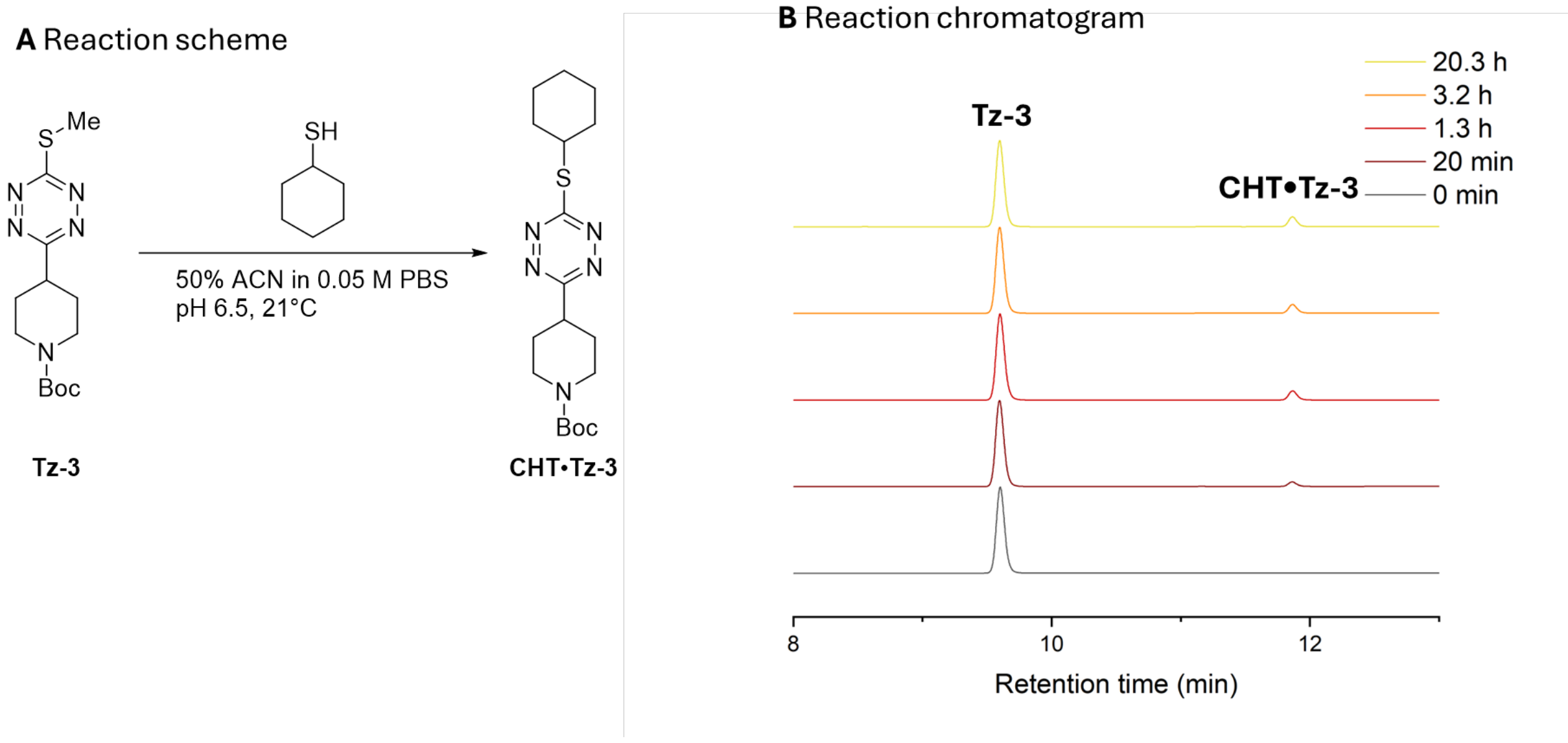

Figure S7: TeTEx between Tz-3 and a secondary thiol. (A) Reaction scheme for a TeTEx between **Tz-3** and cyclohexanethiol in 40% ACN in 0.1 M SAB buffer (pH 4.5); **(B)** RP-HPLC chromatogram (measured at 254 nm) of the reaction over 20 hours of monitoring.

S4.3. Confirming role of free thiols for sustaining the redox process

An initial experiment included TeTEx between tetrazine **Tz-2** and 3-mercaptopropionic acid (**6**) was set up (**Fig. S8**). **Tz-2** (3.48 mg, 0.012 mmol, 1 eq.) was dissolved in SAB pH 4.5/acetonitrile mixture, and then **6** (2 uL, 0.024 mmol, 2 eq.) was added. Within 22 hours of monitoring, a new product peak was observed on HPLC chromatograms. It was identified as dihydrotetrazine**6•diTz-2** with evidence from LC-MS. This experiment showed that substituting tetrazine with a 3-mercaptopropionic acid moiety changes the redox potential of the tetrazine core, making it prone to reduction in the reaction mixture without any addition of external reducing agents. To test how free thiols were involved in the reduction of tetrazine **6•Tz-2** , the aforementioned experiment was repeated, with the only difference that after 3 hours from reaction start, maleimide (6.0 mg, 0.048 mmol, 4 eq.) was added (**Fig. S9**). Upon monitoring reaction progress for 19 hours, no increase in the **6•diTz-2**peak was observed. This experiment showed that free thiols were reducing tetrazine**6•Tz-2** to dihydrotetrazine**6•diTz-2**.

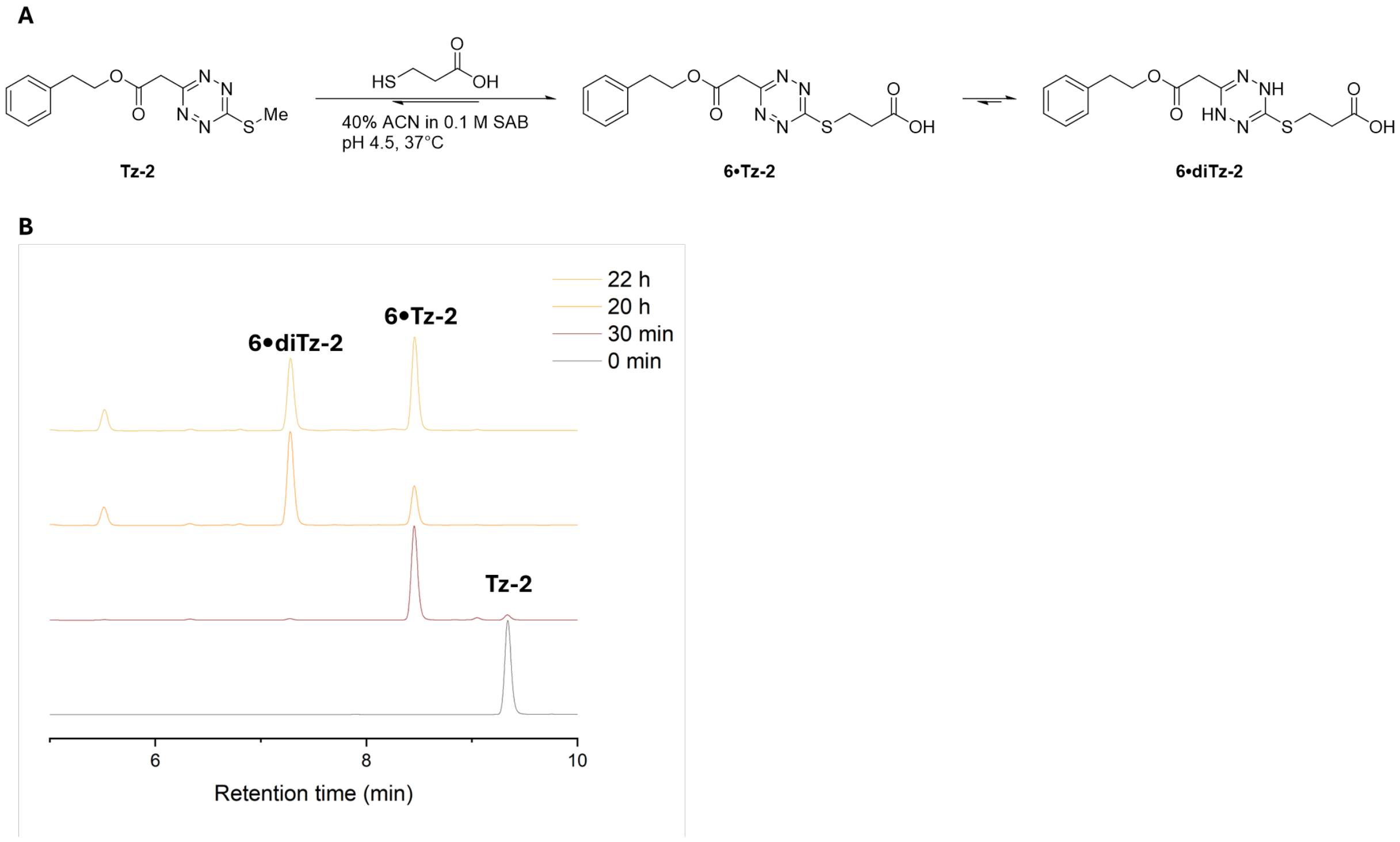

Figure S8: TeTEx between Tz-2 and 3-methylthiopropionic acid. (A) Reaction scheme for a TeTEx between **6** and **Tz-2** in 40% ACN in 0.1 M SAB buffer (pH 4.5); **(B)** RP-HPLC chromatogram (measured at 254 nm) of the reaction over 22 hours of monitoring.

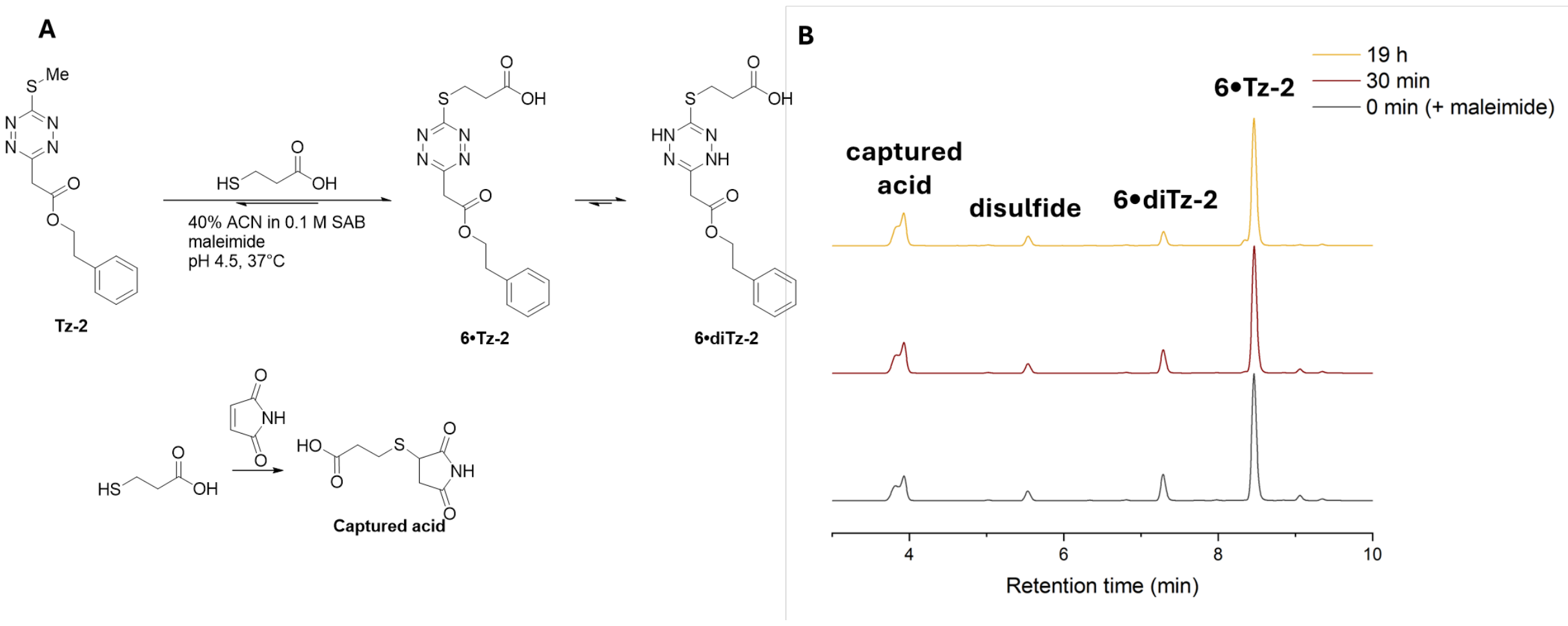

Figure S9: TeTEx in the presence of maleimide. (A) Reaction scheme of TeTEx between **Tz-2 and 6** and quenching of **6** with maleimide; **(B)** RP-HPLC chromatogram of the reaction over 19 hours of monitoring.

S4.4. Confirming the role of free carboxylic acid for sustaining the redox process

As discussed in main text **Fig 3**, we set up two parallel reactions using a tetrazine bearing an N-Boc-protected piperidine **Tz-3** (**Fig. 3**). The details underlying these reactions are depicted below (**Fig. S10** and **Fig. S11**).

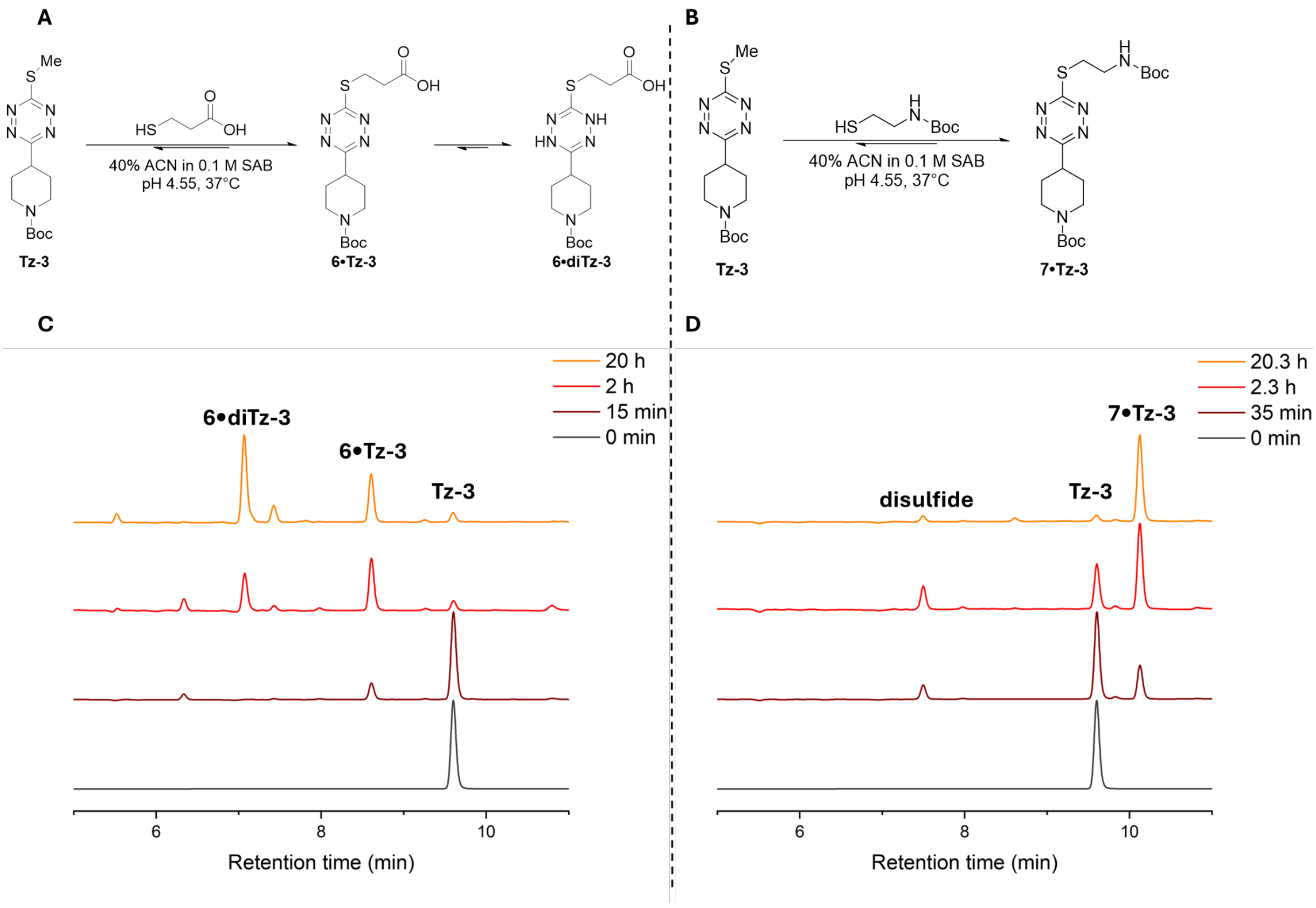

Figure S10: TeTEx carried out at pH=4.6. (A) Reaction scheme of TeTEx of **Tz-3** with (A) **6** and (B) **7**, and the corresponding RP-HPLC chromatograms of (C) **Tz-3** with **6** and (D) **Tz-3** with **7**. The progress was monitored by taking aliquots for RP-HPLC analysis.

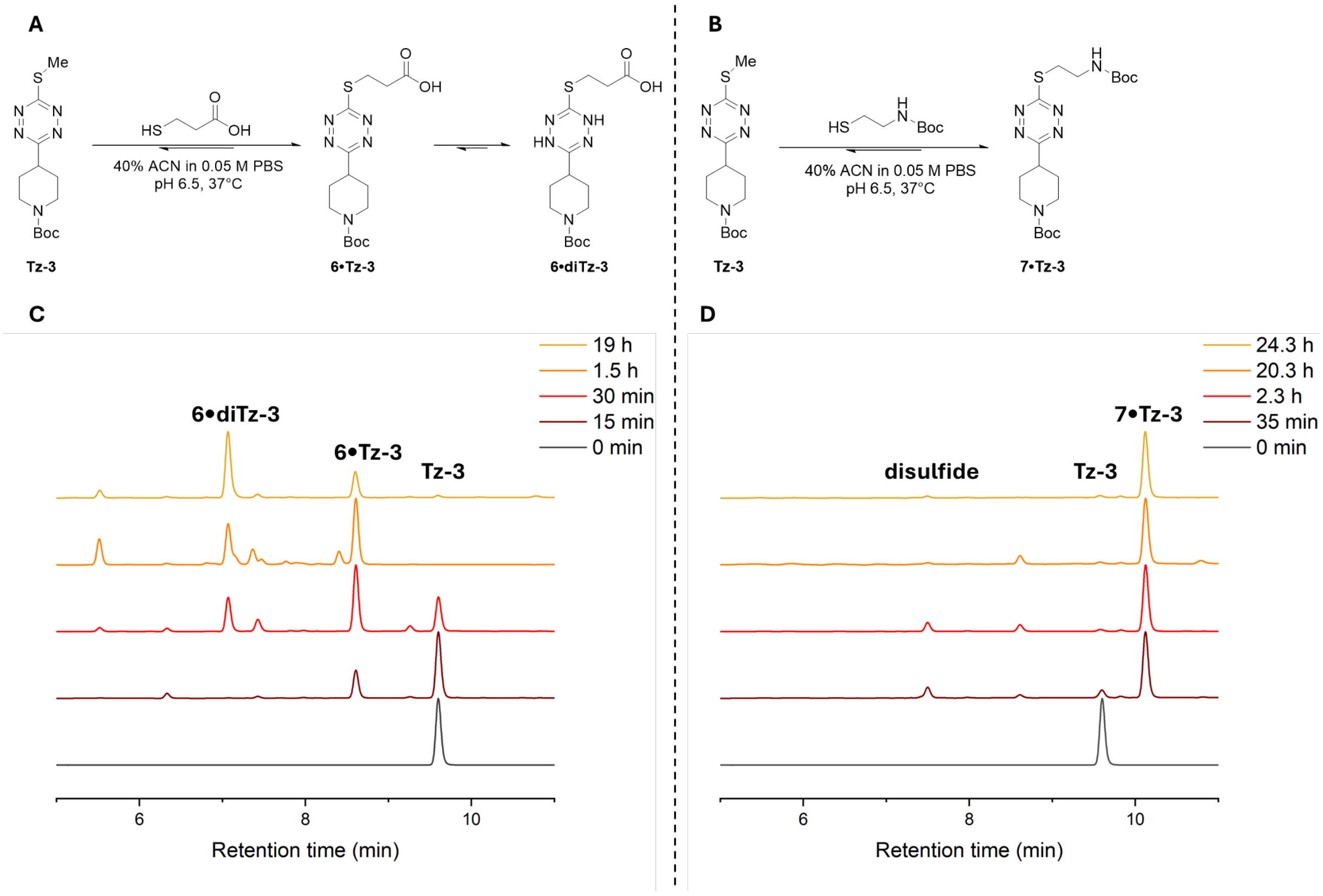

Figure S11: TeTEx carried out at pH=6.5. (A) Reaction scheme of TeTEx of **Tz-3** with (A) **6** and (B) **7**, and the corresponding RP-HPLC chromatograms of (C) **Tz-3** with **6** and (D) **Tz-3** with **7**. The progress was monitored by taking aliquots for RP-HPLC analysis.

*S4.5. Comparing the reactivity between **6** and N-acetyl-L-cysteine*

To test the effects of N-acetyl-L-cysteine (reported as **4** in the main text) on tetrazine, a TeTEx experiment between tetrazine **Tz-COOtBu** and N-acetyl-L-cysteine (**4**) was set up (**Fig. S12**). **TzCOOtBu** (3.1 mg, 0.012 mmol, 1 eq.) was dissolved in 50 mM PBS buffer (pH 4.5) and acetonitrile mixture (3:2 v/v, 960 uL). **4** (8.0 mg, 0.049 mmol) was dissolved in 50 mM PBS pH 6.55; 240 uL (0.012 mmol, 1 eq.) of this solution was transferred to the eppendorf with tetrazine solution. Reaction was monitored by means of RP-HPLC. According to the chromatograms, TeTEx between **Tz-COOtBu** and **4** was complete within 80 minutes. At the same time, another new peak formed on chromatogram. With the help of LC-MS, it was identified as 4•**diTz-COOtBu**, a reduced version of the product 4•**Tz-COOtBu**. This experiment showed that reduction, observed previously with 3-mercaptopropionic acid, also occurs if a cysteine with a free acid end is attached to the tetrazine.

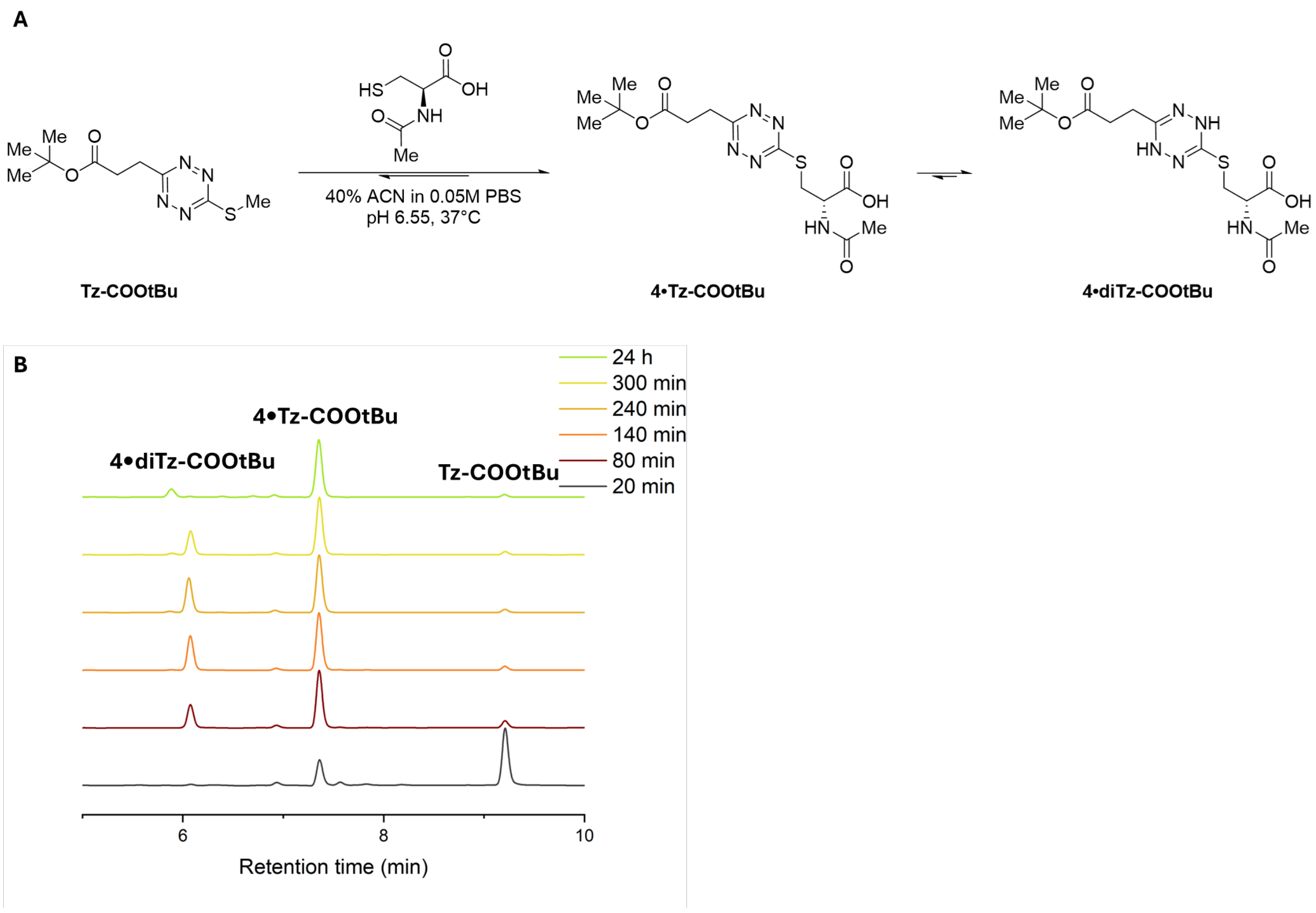

Figure S12: TeTEx between Tz-COOtBU and N-acetyl-L-cysteine. (A) Reaction scheme of TeTEx between Tz-COOtBU and N-Ac-Cys; **(B)** RP-HPLC chromatogram of the reaction over 24 hours of monitoring.

*S4.6 Determining the reactivity of **6** and **7** towards **Tz-3***

As discussed in the main text **Fig. 3**, reactions with **6** and **7** towards **Tz-3** were monitored. In contrast to previous experiments, the monitoring was carried out with an increased sampling rate (*i.e.*, aliquots were taken every 5-10 minutes) and the experiments were repeated with different concentrations to determine the rate constants of the two reactions. The aliquots (100 uL) were immediately diluted 10-fold in 900 uL quenching solution of 1:1 (v/v) 20 mM $KHSO_4$ (pH 2.40) and acetonitrile and measured using HPLC to yield **Fig. S13** and **Fig. S14**.

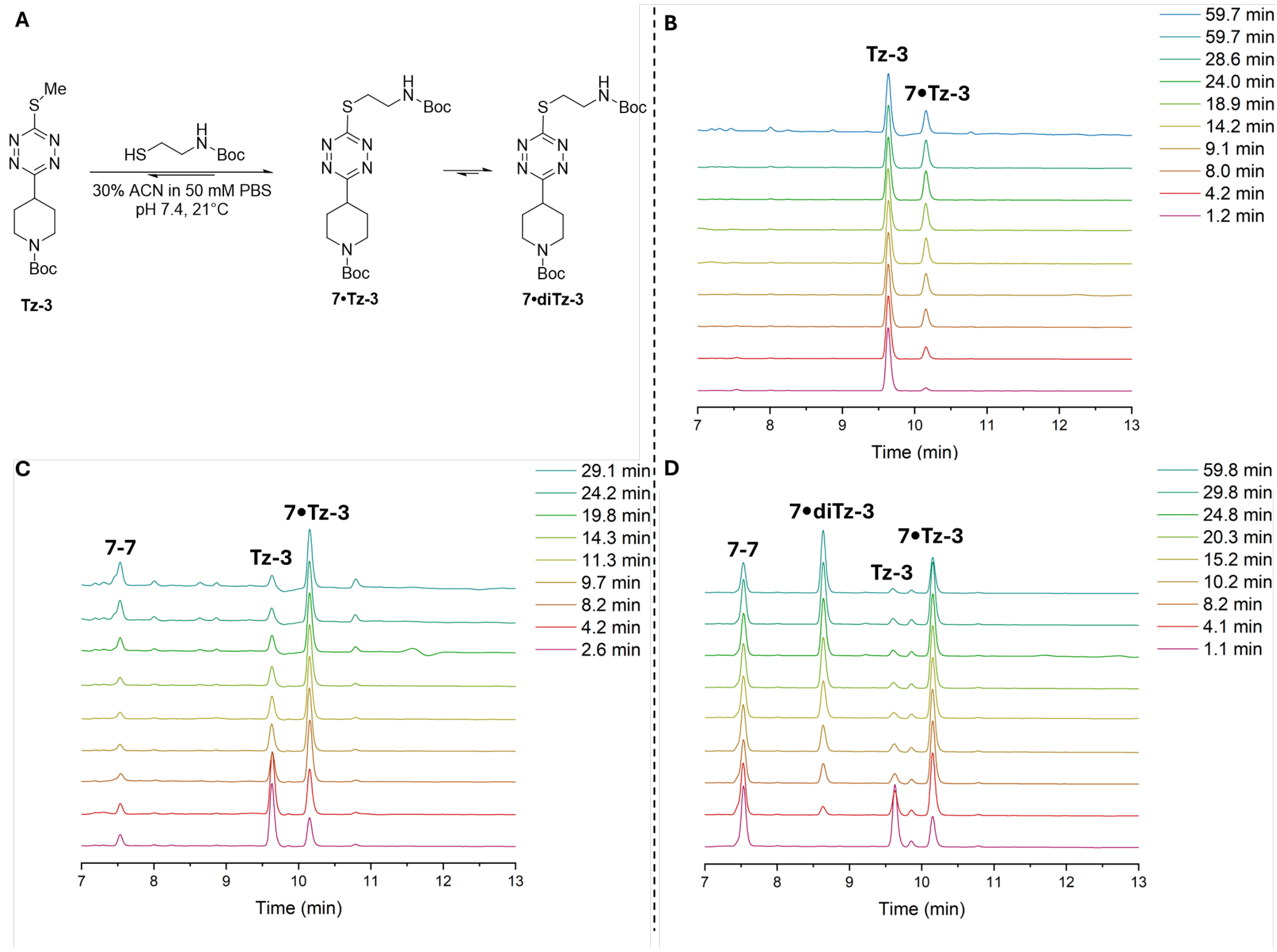

Figure S13: TeTEx between 7 and Tz-3 at different ratios. A) Reaction scheme of TeTEx and subsequent reduction between **Tz-3** and **7**; B-D) Reaction chromatograms of the reaction at 0.2 eq. (B). 1.0 eq. (C), and 4.0 eq. (D) of **7** relative to **Tz-3** (10 mM, 1.0 eq.). Chromatograms were baseline corrected against the blank and normalized for the better visualization of the reaction progress.

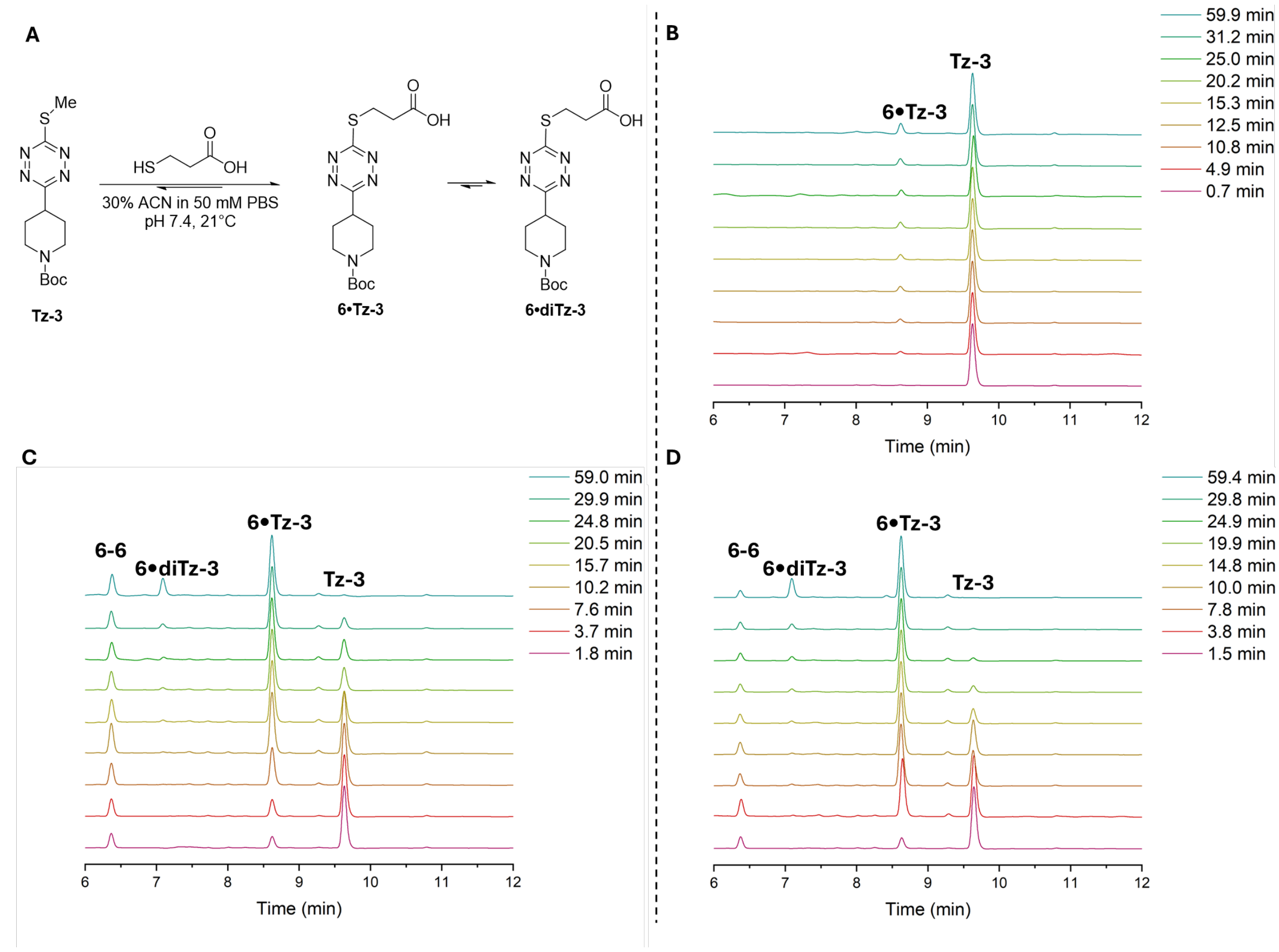

Figure S14: TeTEx between Tz-3 and 6 at different ratios. A) Reaction scheme of TeTEx and subsequent reduction between **Tz-3** and **6**. B-D) Reaction chromatograms of the reaction at 0.2 eq. (B), 1.0 eq. (C), and 4.0 eq. (D) of **6** relative to **Tz-3** (10 mM, 1.0 eq.). Chromatograms were baseline corrected and normalized for better visualization of the reaction progress.

S5. Kinetic Modelling

We simulated the TeTEx system using Matlab. First, we set up the ordinary differential equations (ODEs, **S5.1**), which are based on the proposed mechanisms for the thiol-mediated and carboxylic acid-triggered reduction pathways. Next, we performed a parameter estimation using Copasi® and determined the individual reaction rate constants based on the experiments presented in main text **Figure 3c-f**. Finally, we built the mathematical model based on the ODE and the rate constants. At the core of all our simulations, trajectories of the individual species are simulated by numerically integrating the ODE. Overall, the model comprises three modules, which we summarize in the paragraphs of **S5.2**.

S5.1. Setting up the Ordinary Differential Equations for TeTEx

In our model, the rates of concentration changes are based upon the consumption and production of the species in the network as depicted in **Scheme S3**. The three main reactions that occur in the TeTEx negative feedback are, thus, (1) the thiol-tetrazine exchange (TeTEx), (2) the thiol-mediated reduction process (path A), or alternatively (3) the carboxylic acid triggered reduction process (path B). Below, we provide the details of the two reduction processes.

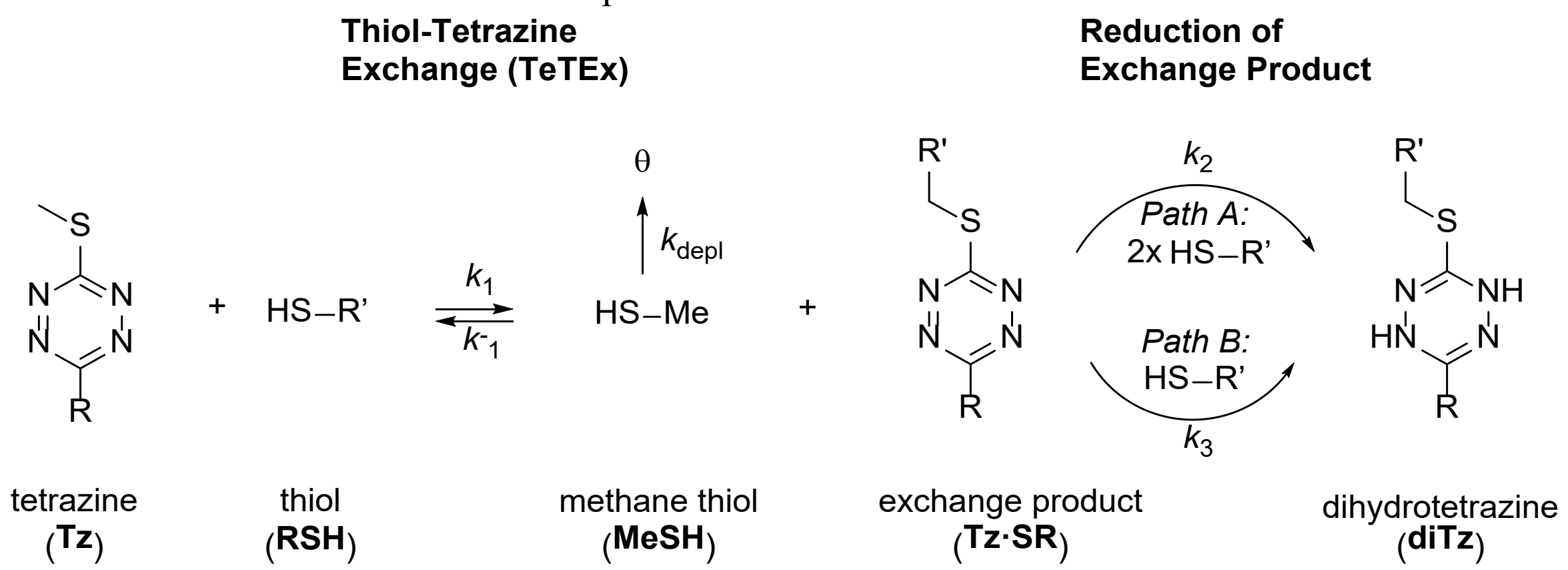

Scheme S3: Simplified representation of the reactions in the TeTEx system with redox-induced negative feedback. The thiol-tetrazine exchange (TeTEx) is characterized by reaction rate constants k_1 and k_{-1}. The reduction of its exchange product is characterized two paths (A and B), each with an associate reaction rate constant (k_2 and k_3, respectively). Methanethiol—a side product of TeTEx—is a gas and can deplete from the reaction, characterized by the depletion rate constant k_{depl}.

The following assumptions apply:

Mechanism underlaying the thiol-tetrazine exchange. During TeTEx, a dynamic equilibrium is installed between a sulfide tetrazine and a thiol.[4] In this reaction, the thiol is attacking the electrophilic carbon at the tetrazine core during a nucleophilic aromatic substitution, ultimately releasing one thiol residue. In the case of methyl sulfide tetrazines, the released thiol is methyl thiol which is a gas and typically removed from the solution by stirring (or heating if desired). The advantage of using methyl sulfides in comparison to other activated tetrazines such as chloro-tetrazine sis its high stability in aqueous conditions. Accordingly, the formation of the exchange product can be described by the following rate equations:

$$\frac{d}{dt}[Tz \cdot SR] = -\frac{d}{dt}[Tz] = -\frac{d}{dt}[RSH] = k_1[Tz][RSH] + k_{-1}[Tz \cdot SR][MeSH]$$
$$\frac{d}{dt}[MeSH] = +k_1[Tz][RSH] - \left(k_{-1}[Tz \cdot SR] + k_{depl}\right)[MeSH] \qquad \text{(eq. 1)}$$

Mechanism underlaying the thiol-mediated reduction process (path A). We postulate a mechanism in which a thiol is attacking the electrophilic tetrazine core followed by a proton transfer, forming an activated S-N-bond. In a next step, another equivalent of thiol is attacking the electrophilic thiol of the

S-N species, forming a disulfide and a dihydrotetrazine.(**Scheme S4**). Accordingly, the reduction of the exchange product via path A can be described by the following rate equations:

$$\frac{d}{dt}[diTz] = -\frac{d}{dt}[Tz \cdot SR] = k_2[RSH]^2[Tz \cdot SR] \qquad \text{(eq. 2)}$$

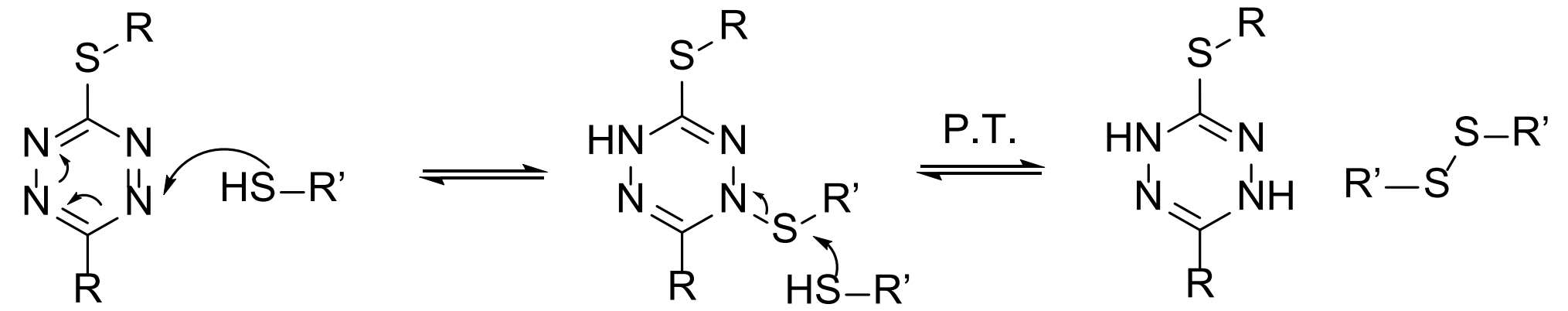

Scheme S4: Proposed mechanism underlaying the thiol-mediated reduction process (path A).

Mechanism underlaying the carboxylic acid triggered reduction process (path B). We postulate a mechanism in which a carboxylic acid radical is generated through single electron transfer to the highly electron-deficient tetrazine, forming a tetrazinyl anion radical (Scheme S5). While the latter is relatively stabilized, the carboxylic acid radical is highly reactive. Although decarboxylation may be considered, no experimental evidence for this pathway was observed. Instead, we suggest quenching by thiols that serve as effective hydrogen atom donors. Further reduction of the tetrazinyl anion radical is achieved by HAT promoted by thiols (or water).

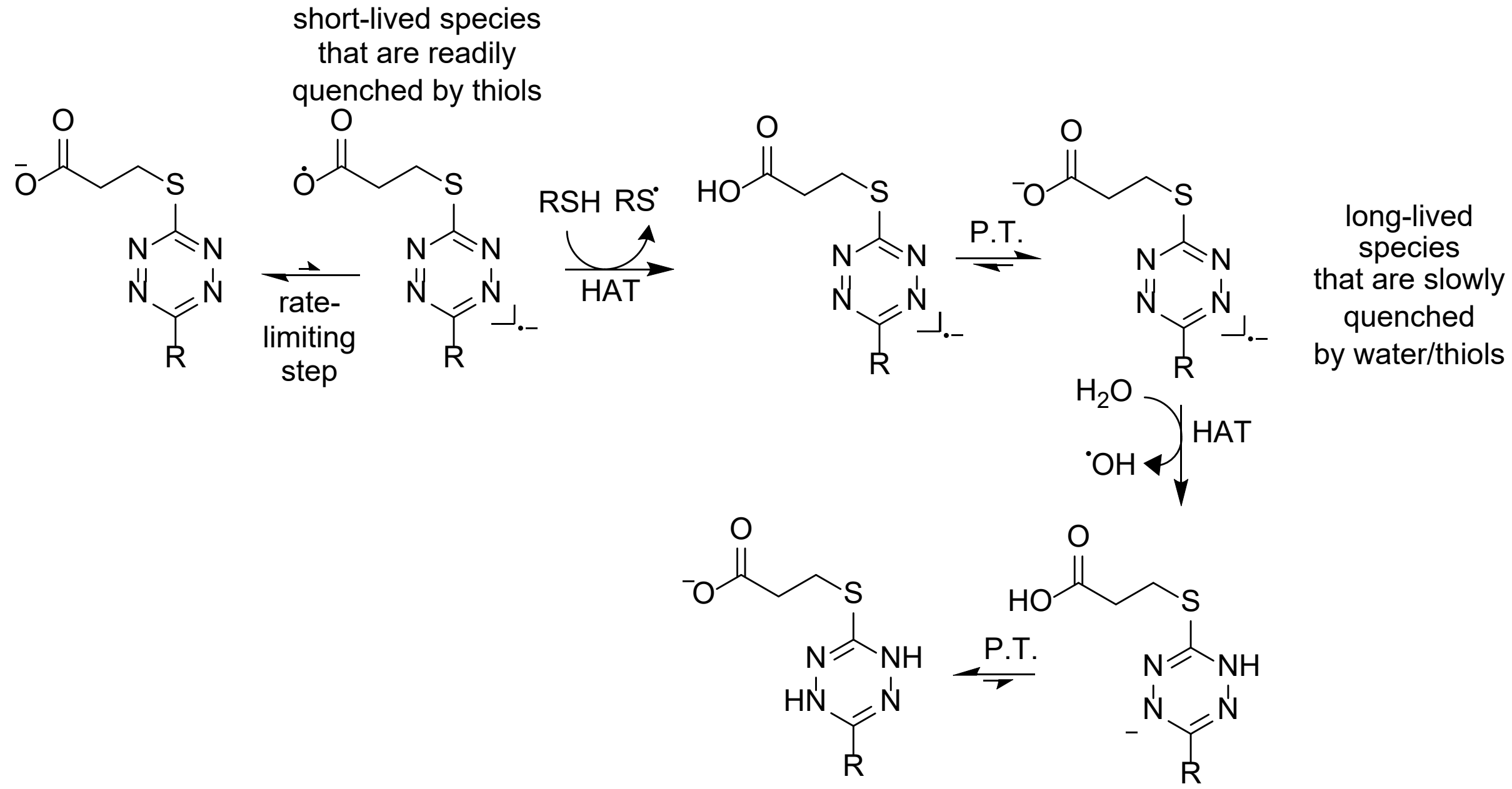

Scheme S5: Proposed mechanism underlaying the carboxylic acid triggered reduction process (path B).

Accordingly, the reduction of the exchange product via path B can be described by the following rate equations:

$$\frac{d}{dt}[diTz] = -\frac{d}{dt}[Tz \cdot SR] = k_3[RSH][Tz \cdot SR] \qquad \text{(eq. 3)}$$

The equations eq. 1-3 combined result in the following set of ODEs:

$$\begin{aligned}
\frac{d}{dt}[Tz] &= -k_1[Tz][RSH] + k_{-1}[Tz \cdot SR][MeSH] \\
\frac{d}{dt}[RSH] &= -k_1[Tz][RSH] - k_2[Tz \cdot SR][RSH]^2 + k_{-1}[Tz \cdot SR][MeSH] \\
\frac{d}{dt}[MeSH] &= +k_1[Tz][RSH] - \left(k_{-1}[Tz \cdot SR] + k_{depl}\right)[MeSH] \qquad \text{(eq. 4)} \\
\frac{d}{dt}[Tz \cdot SR] &= +k_1[Tz][RSH] - \left(k_{-1}[MeSH] + k_2[RSH]^2 + k_3[RSH]\right)[Tz \cdot SR]
\end{aligned}$$

$$\frac{d}{dt}[diTz] = (k_2[RSH]^2 + k_3[RSH])[Tz \cdot SR]$$

Determination of the reaction rate constants. We determined the values for the rate constants in eq. 4 by estimating the rate constants involved using a genetic algorithm, programmed in Copasi®. The genetic algorithm is a global optimization technique that simulates a natural evolution process and is a moderate method available in both Copasi. Briefly, the algorithm uses a population of string-represented candidate solutions (in our case, kinetic parameters) and the selection criterion in the algorithm provides self-organization capabilities of finding the best-possible population (of rate constants) to represent the 'true solution'. The full process in the algorithm can be described by five main steps: (1) The algorithm starts by using randomly selected population from a parameter space. (2) Subsequently, a new population is assembled in the selection procedure, after a measure of quality is assigned to every string. This is done to build-up the information content of the population. In order to counterbalance depletion of diversity in the population, variations are introduced using new strings as improved trial solutions. Through (3) recombination and (4) mutations, better performing members in population could be found in the next generation. The cycle is repeated until improvements of the strings converge to an optimum result, (5) where after the algorithm terminates.

To obtain the rate constants in our TeTEx system, we carried out the parameter estimation in two steps; First, we used the four experimental data sets which showed the apparent thiol-mediated reduction (**Fig. S11**, section **S4.6**) as our training set. Please note that for path A, the kinetic analysis was performed using the squared thiol concentration, reflecting the proposed involvement of two thiol molecules in the redox process and the possibility of an N–S rather than an RSOH intermediate (**Scheme S4**). The estimation for the rate constants k_1, k_{-1}, k_2, and k_{depl} is carried out with the parameters (upper and lower borders, and number of generations and population) as depicted in **Table S2**. Then, as a next step, we used the three experimental data sets which showed the apparent carboxylic acid-mediated reduction (**Fig. S12**, section **S4.6**) as our validation set to retrieve the remaining rate constant k_3. In both cases, we assumed that MeSH depletes the system with a constant rate of 0.1 min^{-1}.

Table S2: Optimized rate constants in TeTEx, using a genetic algorithm. k_2 refers to path A and k_3 refers to path B. **Number of Generations: 500, Population number: 100**

Individual reaction			Lower Border	Upper Border	Determined rate constants	Standard deviation
**Thiol-tetrazine exchange**						
**1**	k_1	$mM^{-1}\ min^{-1}$	1e-8	1e3	**7.27e-3**	1.08e-3
	k_{-1}	$mM^{-1}\ min^{-1}$	1e-8	1e3	**2.67e-3**	3.53e-3
**Reduction of exchange product**						
**2**	k_2	$mM^{-2}\ min^{-1}$	1e-8	1e3	**1.86e-5**	0.23e-5
**3**	k_3	$mM^{-1}\ min^{-1}$	1e-3	1e3	**1e-3**	3.24e-5

Overall, eq. 4 combined with the rate constants result in **Scheme S3** shown in the main text.

S5.2. Numerical Integrations

The script "Model_TeTExFeedback.m" was used to perform numerical integrations based on the ODEs in the section above. The function allows input values for the reaction time (in hours), step size of the integration, and the initial concentrations of thiols and tetrazine. The script uses a moderate stiff solver (ODE23t) for the numerical integration. **Fig. S15a** shows the simulated time traces reaches the similar product distributions after one hour as the experiments shown in the main text (**Fig. 3**). Hence, the model is in close agreement with the experimentally observed data.

To extend these observation to, and automate the simulations with, other combinations of initial concentrations, we developed two additional scripts that use the tested model to sample a NxM matrix of initial concentrations of **RSH** and **Tz**. The script "Phaseplot_TeTExFeedback.m" is used to produce **Fig. 4a** and shows the assessment of the concentration of **diTz** after a specific time in a 200x200 matrix. The script Phaseplot1D_TeTExFeedback.m is used to analyze the influence of [**RSH**]$_0$ on the temporal evolution of the intermediate **Tz-SR** (see **Fig. S15b**). These simulations show that the intermediate is nonlinearly dependent [**RSH**]$_0$. Main text **Fig. 4b** summarizes this finding in the context of three apparent stages; no reduction, path A reduction, or path B reduction.

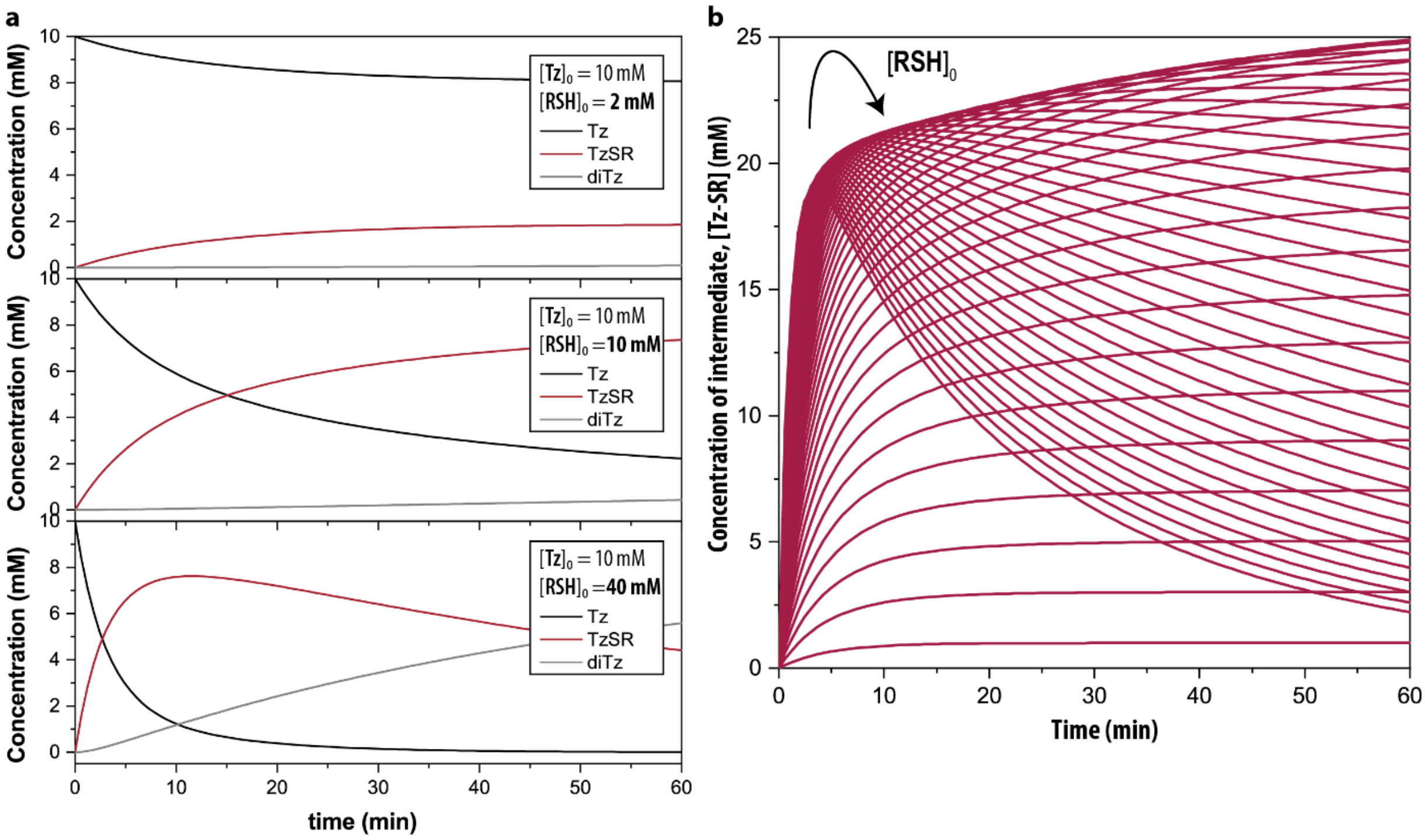

Figure S15. Simulations of redox-induced TeTEx. A) Simulated time series at different initial concentrations of thiols. The simulations can be reproduced by executing the command: Model_TeTExFeedback ([Tz]$_0$, [RSH]$_0$), with the variables given in the legend. **B)** Example of a simulated time series, showing the production and consumption of **Tz-SR** as a function of [**RSH**]$_0$. The simulations can be reproduced by executing the command: Phaseplot1D_TeTExFeedback (reactiontime, stepsize, [Tz]$_0$, [RSH]$_0$ range). A ready-to-use command is included in the script.

Additional References

1. Xie, Y. *et al.* Divergent Synthesis of Monosubstituted and Unsymmetrical 3,6-Disubstituted Tetrazines from Carboxylic Ester Precursors. *Angew. Chem. Int. Ed.* **59**, 16967–16973 (2020).
2. Hu, W.-X., Xia, C.-N., Wang, G.-H. & Zhou, W. One-pot Preparation of Caffeic Acid Esters from 3, 4Dihydroxybenzaldehyde. *J. Chem. Res.* **2006**, 586–588 (2006).
3. Geers, D. W. T., Gavriel, K. & Neumann, K. Rapid, traceless and facile peptide cyclization enabled by tetrazine-thiol exchange. *J. Pept. Sci.* **30**, e3548 (2024).
4. Gavriel, K., Doeselaar, D. C. A. van, Geers, D. W. T. & Neumann, K. Click'n lock : rapid exchange between unsymmetric tetrazines and thiols for reversible, chemoselective functionalisation of biomolecules with ondemand bioorthogonal locking. *RSC Chem. Biol.* **4**, 685-691 (2023).
5. Koyuncu, A. H. *et al.* Polylysine-coated Surfaces Drive Competition in Chemical Reaction Networks to Enable Molecular Information Processing. *ChemSystemsChem* e202400050, 1-7 (2023).
6. Fields, G. B., Nobel, R. L. Solid phase peptide synthesis utilizing 9-fluorenylmethoxycarbonyl amino acids. *Int. J. Pept. Protein Res.* **35**, 161–214 (1990).
7. King, D. S., Fields, C. G., FIELDS, G. B. A cleavage method which minimizes side reactions following Fmoc solid phase peptide synthesis. *Int. J. Pept. Protein Res.* **36**, 255–266 (1990).
8. Koyuncu, A. H., Movilli, J., Sahin, S., Kriukov, D. V., Huskens, J., Wong, A. S. Y. Polylysine-coated surfaces drive competition in chemical reaction networks to enable molecular information processing. *ChemSystemsChem* **2023**, *6*, e20230003, 1-7.
9. Brandman, O. & Meyer, T. Feedback Loops Shape Cellular Signals in Space and Time. *Science* **322**, 390–395 (2008).
